# Solution Phase Protein Dynamics: Influences of ESI Buffers on Hydration

**DOI:** 10.1021/acs.analchem.6c02162

**Published:** 2026-08-10

**Authors:** Emily Burningham, Carter Lantz, Robert L. Rider, Syuan-Ting Kuo, Sangho D. Yun, Arthur Laganowsky, David H. Russell

**Affiliations:** Department of Chemistry, 14736 Texas A&M University, College Station, Texas 77843, United States

## Abstract

Buffers are commonly selected for their compatibility with biochemical measurements or their specific capabilities, yet buffer interactions with proteins and the surrounding water can actively reshape protein activity, structure, and dynamics. Here, we compare the influences of three widely used electrospray ionization (ESI) buffers on the dynamics and stability of wild-type transthyretin (wtTTR) and TTR mutants (V30M, L55P, T119M, V122I) using native mass spectrometry (nMS). Ammonium acetate (AmAc), ethylenediammonium diacetate (EDDA), and triethylammonium acetate (TEAA) are commonly employed in nMS because they stabilize the sample in solution, facilitate gentle ionization, and minimize adduct formation on proteins and protein complexes. In comparison to AmAc, EDDA and TEAA reduce the average charge state (Z_avg_) of ions, consistent with conformational changes that decrease the solvent-accessible surface area (SASA) of proteins. Intact protein hydrogen–deuterium exchange experiments indicate significantly lower deuterium uptake for all TTR proteoforms in EDDA compared with AmAc or TEAA. Ion mobility MS revealed that each TTR proteoform has a larger average CCS and broader CCS distributions in AmAc, indicating greater conformational heterogeneity and dynamics than in EDDA or TEAA. Measurements of TTR tetramer disassembly and reassembly further demonstrated that the buffer identity strongly influences tetramer stability in solution. The buffer-dependent effects arise from differences in buffer-protein and buffer-solvent interactions that alter the hydration of the protein. These results highlight that buffer composition can significantly influence experimentally observed protein dynamics and stability, with important implications for interpreting measurements across biochemical and biophysical techniques.

## Graphical abstract

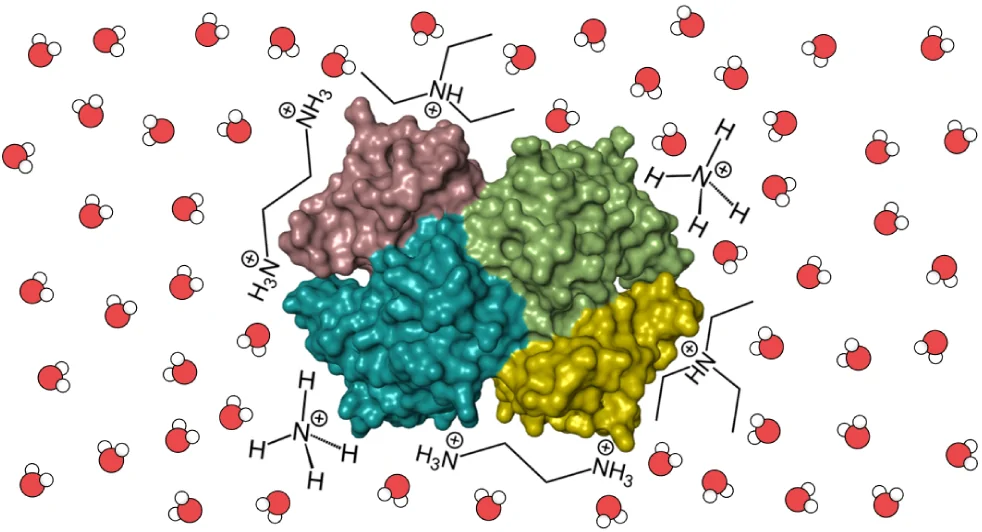

## Introduction

Unraveling the influences of the solution environment on the structure, stability, and dynamics of proteins and protein complexes is a daunting task. Protein structures determined by X-ray crystallography and cryo-electron microscopy are often correlated to solution structure, but are complexes trapped in a crystalline lattice representative of hydrated complexes? ^1,2^ Water plays a fundamental role in the physicochemical properties of proteins and should not be overlooked.^3^ The electrospray ionization (ESI) process results in “freeze-dried” biomolecules that capture the proteins’ solution-phase structure and dynamics.^4–6^ Through optimization of ESI, native mass spectrometry (nMS) emerged as a powerful technique for protein analysis and is often coupled with multiple techniques to capture dynamic structures.^7,8^ Techniques paired with nMS to further analyze proteins include hydrogen–deuterium exchange (HDX) for identifying protein–protein or protein–ligand interactions along with protein dynamics in solution,^9–12^ and ion mobility (IM) for additional ion separation and structure comparison in the gas phase.^5,13–16^

Native mass spectrometry utilizes volatile buffers such as ammonium acetate (AmAc), ethylenediammonium diacetate (EDDA), and triethylammonium acetate (TEAA) for sample ionization. Alkylammonium ions, such as EDDA and TEAA, along with many naturally occurring osmolytes, act as effective charge-reducing reagents, producing ions with lower average charge states (Z_avg_) than those generated from the most commonly used nMS buffer, AmAc.^17–20^ Changes in Z_avg_ have been attributed to differences in the solvent-accessible surface area (SASA) of proteins, which alter the number of sites available for protonation during ESI.^21^ Accordingly, higher Z_avg_ values are generally associated with a more unfolded or dynamic structure, whereas lower Z_avg_ values are indicative of a more compact or less dynamic structure.^22–24^ Alternatively, the proton affinity and gas-phase basicity (GB) of buffers have also been proposed to influence Z_avg_.^25–29^ The AmAc ammonium ion is thought to facilitate ionization by transferring a proton to the protein before departing as ammonia,^30^ whereas the higher basicities of EDDA and TEAA promote charge reduction by the transfer of protons from the protein to the more basic buffer, resulting in a lower charge.^28^ Rather than being governed by either mechanism alone, the observed Z_avg_ likely reflects a balance between protein SASA and buffer basicity.^25,26^ While EDDA and TEAA are recognized as charge-reducing agents,^17,18^ it remains unclear whether these alkylammonium ions also influence the physicochemical properties of proteins in solution.

Proteins in solution are dynamic macromolecules that constantly interact with both themselves and the surrounding environment. Protein–water interactions (hydration) and protein–buffer interactions play a central role in regulating protein conformational entropy, stability, ligand binding, and dynamics.^31–38^ Consistent with this view, Campanile and Graziano presented compelling evidence that “water is the engine of protein folding.”^39^ Hydration can be altered by modulating solution conditions (e.g., ionic strength, temperature, pH, and the presence of cosolvents or other species/molecules),^17,37,40–48^ as well as through modifications to the protein itself, such as missense mutations or ligand binding.^23,42,49–51^ These shifts in hydration can directly influence protein stability or protein–ligand binding affinity and could be attributed to more chaotropic (disordered hydration) or kosmotropic (ordered hydration) behavior.^3,52^ Darby et al. reported a >1000-fold decrease in binding affinity following a single missense mutation, despite the residue making no direct contact with the ligand.^50^ Similarly, nMS studies showed that ADP and ATP ligand binding to GroEL differed in EDDA compared to AmAc and TEAA.^42,53^ Additional work combining nMS with ion mobility MS (IM-MS) demonstrated that common biological buffers alter the conformational distribution of α-synuclein,^54^ while another study found that both nMS and biological buffers influence the enzymatic activity of the cysteine desulfurase, IscS.^6^ Collectively, these studies highlight that buffers can substantially influence the behavior and physicochemical properties of proteins by altering hydration.

Transthyretin (TTR) is a 56 kDa homotetramer that transports thyroxine and retinol in the human body.^55,56^ Wild-type TTR and various TTR proteoforms have been linked to transthyretin amyloidosis (ATTR), a life-threatening disease catalyzed by misfolded TTR monomers following tetramer dissociation.^57–60^ TTR stability and aggregation propensity are greatly influenced by solution conditions such as pH and ionic strength,^61–63^ both of which alter hydration.^47^ Lantz et al. highlighted that the disruption of conserved interfacial water molecules plays a central role in tetramer destabilization.^23,49^ Additionally, the presence of D_2_O was shown to shift the bulk hydration of TTR, affecting its structure and stability.^23^ Because of its sensitivity to hydration, TTR and its mutants (V30M, L55P, T119M, V122I) are promising models for investigating how AmAc, EDDA, and TEAA influence protein behavior in solution.

This study investigates the effects of AmAc, EDDA, and TEAA on TTR stability and dynamics using intact protein HDX-MS and IM-MS. Intact protein HDX-MS experiments reveal varying levels of deuterium uptake across buffers, while IM-MS experiments show buffer-dependent differences in average collision cross section (CCS_avg_) and conformer abundances of TTR. Intact protein HDX-MS further enables direct observation of TTR tetramer disassembly and reassembly in solution, demonstrating that EDDA can suppress tetramer disassembly compared to AmAc or TEAA. Collectively, these data indicate that nMS buffers interact with the protein and alter its hydration, therefore directly influencing protein dynamics and physicochemical properties.

## Methods

### TTR Expression, Buffer Preparation, and Sample Preparation

TTR was expressed and purified as previously described.^64^ The AmAc and EDDA solutions in H_2_O were prepared by dissolving the salts in LC–MS H_2_O to 200 mM; the pH was then adjusted to 7.0 with 28% ammonium hydroxide. One solution of 67 mM EDDA was prepared the same way for a control experiment comparing the ionic strengths of AmAc and EDDA. For TEAA, 1 M TEAA in H_2_O was diluted to 200 mM with LC–MS H_2_O, and pH was adjusted as needed with either 28% ammonium hydroxide or LC/MS grade Optima acetic acid (Fisher Chemical, Ottawa, ON). For the D_2_O buffers, AmAc and EDDA were dissolved first with 99.9 atom % D_2_O and then H_2_O to have a final percentage of 80% D_2_O in solution. For the TEAA buffer in D_2_O, 1 M TEAA in H_2_O was diluted to 200 mM with 99.9 atom % D_2_O. All D_2_O buffers were adjusted to a pH_read_ of 7.0 with 28% ammonium hydroxide or glacial acetic acid, as necessary.

For intact protein HDX-MS experiments, TTR samples were diluted to 40–50 μM with 1 M AmAc and treated with 10% volume of 3 mg/mL diethylenetriaminepentaacetic acid (DTPA) and incubated at ambient temperature for 15 min. Afterward, 250 μM of *N*-ethylmaleimide (NEM) was added to the sample and then incubated at ambient temperature for an additional 30 min. The sample was buffer exchanged into AmAc, EDDA, or TEAA (H_2_O) buffer solutions using Bio-Spin 6 SEC columns (Bio-Rad, Hercules, CA). The HDX reaction was initiated by diluting the TTR sample 10-fold with the 80% D_2_O version of the buffer of choice, resulting in a 70% D_2_O solution and 4–5 μM TTR.

For ion mobility experiments, TTR aliquots were diluted to 20 μM with 1 M AmAc and 10% volume of 3 mg/mL DTPA and incubated for 15 min. This was later buffer exchanged into 200 mM AmAc, 200 mM EDDA, and 200 mM TEAA (H_2_O, pH 7.0). AmAc, EDDA, and TEAA were all purchased from Sigma-Aldrich (St. Louis, MO).

### Intact Protein Hydrogen–Deuterium Exchange

Products from intact protein HDX experiments were analyzed using an ultrahigh mass range (UHMR) mass spectrometer (Thermo Fisher Scientific, Bremen, Germany). Samples were electrosprayed directly into the UHMR via gold-coated pulled borosilicate capillaries made with a P-1000 micropipette puller (Sutter Instrument, Novato, CA) and coated with a Leica EM ACE 200 sputter coater (Leica Microsystems, Wetzlar, Germany). The HDX reaction was monitored starting at 2 min and ending after 96 h; no quenching of the reaction was performed. After initial time points were taken (2–60 min), samples were incubated between time points at 25 °C using Fisherbrand Mini Dry Bath (Fisher Scientific, Waltham, MA). Percent hydrogen exchange was determined based off the total hydrogen atoms available for exchange, which were calculated according to the protein sequence. Instrument parameters were set at HCD cell trapping gas pressure of 4.0, 3,000 resolution for V30M, and 6,000 resolution (at 400 *m*/*z*) for all other TTR proteoforms. V30M was electrosprayed at 3,000 resolution due to the presence of multiple adducts that made tracking HDX more difficult. Twenty-five eV in-source CID energy was used along with 55–120 V HCD energy, −10 V in-source trapping, and a spray voltage ranging from 0.80 to 1.40 kV. Deconvoluted mass was obtained using UniDec.^65^ The biexponential fit of the HDX data was determined using the ExpGro2 function in the Speedy Fit app in OriginPro (OriginLab, Northampton, MA).

The mole fraction of the intact vs reassembled tetramer was calculated using the relative intensity values of the initial tetramer peak in the mass spectra to that of the second, higher mass peak using values obtained from UniDec.^65^ The graphic in Figure 4, depicting the tetramer disassembly and reassembly, was created in BioRender. Burningham, E. (2026) https://biorender.com/zrc7q4h.

### L55P NEM Subunit Exchange Experiment

Two samples of L55P were treated with DTPA as mentioned in the sample preparation section. NEM was added to one sample (NEM-bound) but not the other (apo). After incubation, samples were buffer exchanged using Bio-Spin 6 SEC columns (Bio-Rad, Hercules, CA). The NEM-bound L55P was incubated at room temperature overnight before initiating experiments. Both NEM-bound and apo samples were diluted to 5 μM with 200 mM AmAc, pH 7.0 (H_2_O), and mixed in a 1:9 ratio of apo:NEM-bound to have similar intensity apo and NEM-bound peaks in the mass spectra. L55P was electrosprayed using gold-coated borosilicate capillaries onto an exactive plus extended mass range (EMR) Orbitrap mass spectrometer (Thermo Fisher Scientific, Wetzlar, Germany). After mixing, the reaction was monitored over a period of 96 h. The sample was incubated at 25 °C between collection times using a Fisherbrand Mini Dry Bath. Instrument parameters include HCD cell trapping gas pressure of 5.0, 17,500 resolution (at 400 *m*/*z*), 30 V HCD energy, and spray voltage ranging from 0.90 to 1.30 kV.

### Ion Mobility Mass Spectrometry and CCS_avg_ Calculations

IM-MS experiments were conducted on an Agilent 6560 IM-QTOF mass spectrometer and analyzed with Agilent Hunter IM-MS Browser (Agilent Technologies, Santa Clara, CA). The sample was electrosprayed with the same gold-coated capillaries described above. Instrument parameters include a spray voltage of 1450 V, 250 °C drying gas temperature, 5 L/min drying gas flow rate, 300 V fragmentor voltage, and 50 V collision cell voltage. Spectra were collected using the 3-bit multiplexing feature and demultiplexed with PNNL PreProcessor.^66^ Drift time was calculated to CCS using the method described by Stow et al.^67^ CCS_avg_ was calculated by first deconvoluting and calculating peak area for all conformers present across all charge states in the CCS profile. This was done using raw intensity values extracted from Agilent IM-MS Browser and fitted with OriginPro 2025. A weighted average of all conformers based off peak area was calculated to determine CCS_avg_ using the following formula, where *W* represents the weighted CCS_avg_, *w*_*i*_ is the intensity of the peaks, and *X_i_* is the CCS value:

1 $$W=\frac{\overset{n}{\underset{i=1}{\sum }}w_{i}\cdot X_{i}}{\overset{n}{\underset{i=1}{\sum }}w_{i}}$$

The average and standard deviation of the CCS_avg_ values were subsequently calculated across 3 trials. The cumulative fit for CCS_avg_ plots was performed by summing up the raw intensities of the interpolated CCS values for all charge states. For each trial, all intensity values were normalized to where the highest intensity value for the cumulative fit was equal to 1, after which the average and standard deviation were calculated from 3 trials.

## Results and Discussion

Compared to AmAc, EDDA and TEAA are charge-reducing buffers that also influence ligand binding affinities and thermodynamics, viz., enthalpy–entropy compensation.^42,53,68^ Here, we investigate additional influences of these buffers using intact protein HDX-MS and IM-MS. Intact protein HDX-MS provides insight into the dynamics of proteins by comparing the number of hydrogen atoms that are replaced by deuterium atoms in solution.^12^ Complementary dynamics information is obtained via IM-MS by separating ions in the gas phase according to their size and shape, where the rotationally averaged surface area of the ion, i.e., collision cross section (CCS), can be calculated.^13,15^ Together, these techniques show that the ESI buffers AmAc, EDDA, and TEAA influence the stability, dynamics, and hydration of the TTR complex.

### TTR Solvent Accessibility Varies in AmAc, EDDA, and TEAA

Intact protein HDX-MS was used to assess how the nMS buffers AmAc, EDDA, and TEAA affect the physicochemical properties of TTR in solution. These experiments were extended to TTR mutants V30M, L55P, T119M, and V122I to further evaluate how these mutations may influence TTR stability and dynamics (Figure 1). Percent deuterium exchange (%D) was calculated based on the total number of exchangeable hydrogens on the TTR tetramer and was monitored over 96 h (see Supporting Information, Figure S1). Throughout the 96 h, wild-type TTR (wtTTR) exhibits similar HDX profiles in AmAc and TEAA; however, the %D was significantly less in EDDA (Figure 1A). The trend of decreased %D in EDDA compared to AmAc or TEAA was observed for all proteoforms of TTR analyzed (Figure 1B–F; Figure S1). Interestingly, V30M was the only TTR proteoform that exhibited less %D in TEAA relative to AmAc, especially at the initial time points (Figure 1B). However, the overarching observation still holds true that EDDA significantly decreases the %D of TTR.

**Figure 1:**
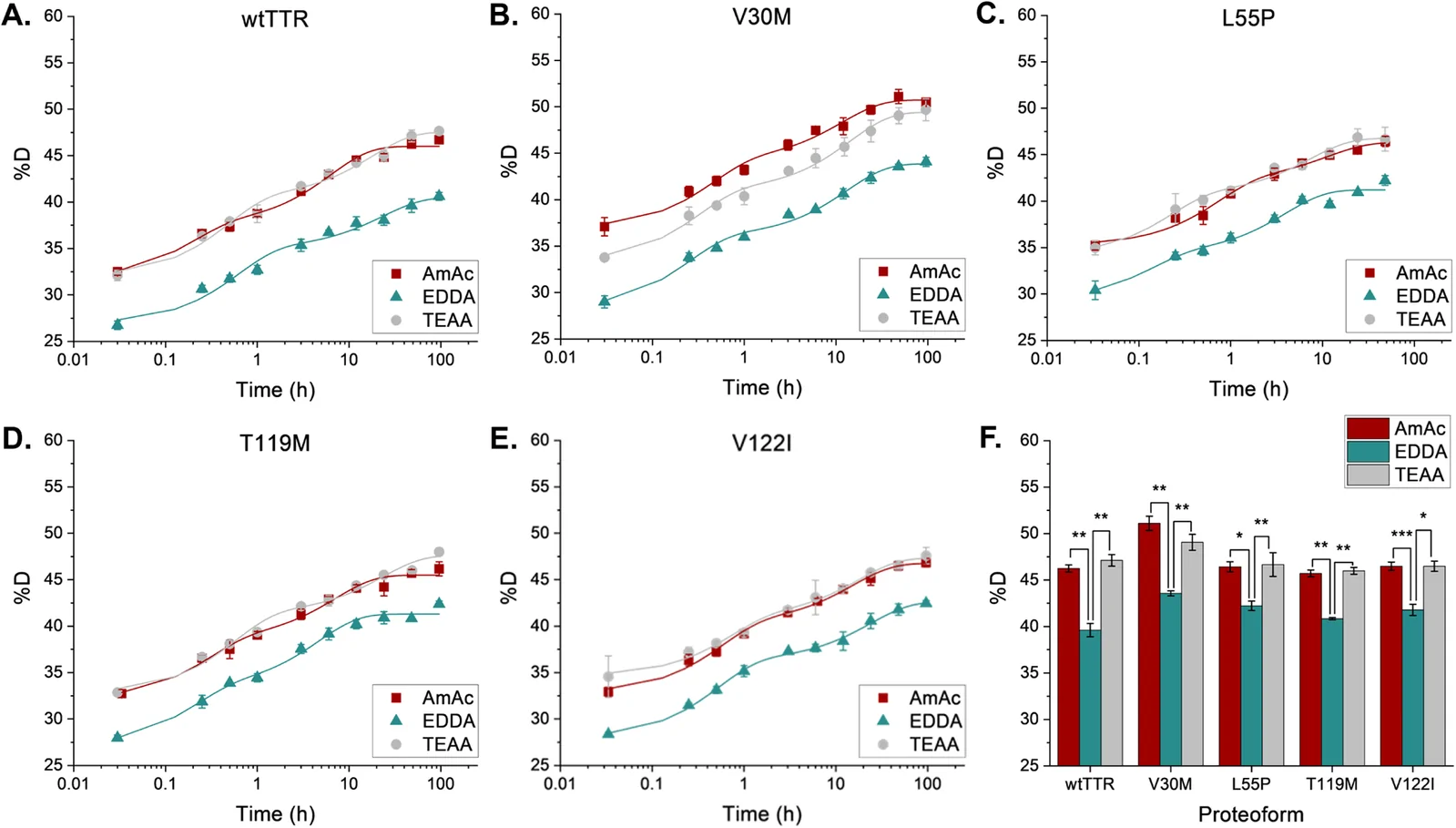
Percent deuterium (%D) relative to total exchangeable hydrogens for wtTTR **(A.)**, V30M **(B.)**, L55P **(C.)**, V122I **(D.)**, and T119M **(E.)**, fitted with a biexponential exchange function. The *x*-axis was plotted on a logarithmic scale. Reported are the mean and standard deviation (*n* = 3). Data for L55P are not reported after 48 h due to tetramer instability. **(F.)** %D at 48 h for all TTR proteoforms and buffers. Across all samples, TTR exhibits a significant decrease in exchange in EDDA compared to AmAc or TEAA. Statistical significance is indicated as *p < 0.05, **p < 0.01, ***p < 0.001 (paired two-sample *t*-test, *n* = 3).

There are multiple elements of the protein structure that could affect HDX. Two of these elements are 1) solvent accessibility of the residues with exchangeable hydrogen atoms and 2) the existence of hydrogen bond networks throughout the protein.^12,69,70^ The decrease in the %D of TTR in EDDA (Figure 1) indicates that fewer hydrogen atoms are available for exchange. The EDDA cation (ethylenediammonium) differs structurally from the AmAc (ammonium) and TEAA (triethylammonium) cations in that the alkylamine is composed of two amino groups capable of interacting with the protein simultaneously (Table 1). Previous studies on the SR1 mutant of GroEL concluded that EDDA interacts more strongly with the protein compared to ammonium or triethylammonium ions.^53^ The results shown here further support this observation. Stronger EDDA-protein interactions could prevent exchangeable sites on the protein from interacting with the surrounding D_2_O molecules and lower the %D observed in EDDA. EDDA could also be creating a more ordered hydration shell that restricts movement, thus leading to lower %D. Shifts in interactions with the solvent (D_2_O) would also restructure the hydration of the protein and thus its physicochemical properties.

**Table 1:**
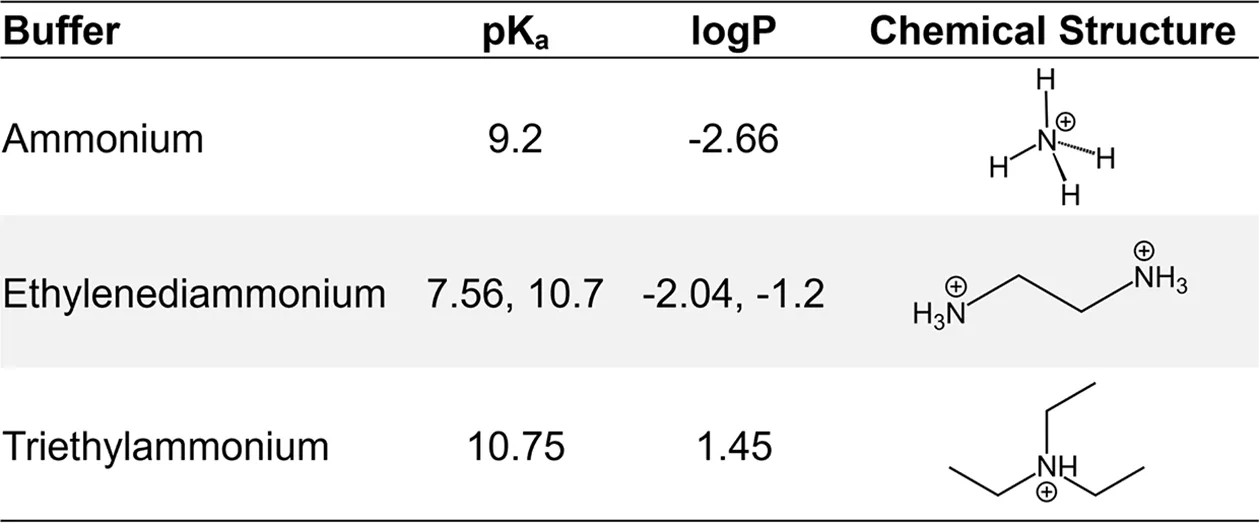
pK_a_, log *P* Values, and Structures of Ammonium, Ethylenediammonium, and Triethylammonium as Reported by PubChem Database

Although TTR largely exhibited similar %D in AmAc and TEAA, this does not mean that they interact with the protein in the same way. One piece of evidence supporting this is that V30M experiences less %D in TEAA compared with AmAc. Additionally, the AmAc cation has a partition coefficient (log *P*) of −2.66, indicating that AmAc has hydrophilic tendencies, while the TEAA cation has a log *P* of 1.45, indicating that TEAA has more hydrophobic tendencies. Both the AmAc and TEAA cations would be protonated at neutral pH, but their structures are very different (Table 1). The cation in AmAc is exposed, allowing it to interact readily with the charged residues on the protein. In comparison, the TEAA cation has three hydrophobic ethyl groups that could deter or weaken TEAA interactions with negatively-charged residues on the protein. Since the log *P* of the TEAA cation indicates hydrophobic tendencies, it is likely that TEAA will interact more readily with hydrophobic residues on the protein compared to AmAc. Thus, despite similar overall HDX, AmAc and TEAA will interact with the protein in their own distinct ways, thereby altering the hydration and dynamics of TTR.

Since the ionic strength of EDDA is approximately three times that of AmAc,^18^ we examined whether the reduced exchange observed in EDDA was due to its ionic strength rather than an effect from EDDA itself. An intact protein HDX-MS experiment was performed in 67 mM EDDA and showed that although wtTTR %D in 67 mM EDDA was higher than in 200 mM EDDA, %D was still lower than in 200 mM AmAc (Figure S2). These data support the conclusion that EDDA alters protein physicochemical properties by altering hydration and that ionic strength alone cannot account for the significantly lower %D compared to AmAc or TEAA.

### ESI Buffers Influence the Average CCS and Dynamics of TTR

IM-MS experiments were performed on TTR to better understand how AmAc, EDDA, and TEAA influence the dynamics of TTR and, consequently, its collision cross section (CCS). The average CCS (CCS_avg_) of TTR was determined (see Methods) to evaluate the overall size and dynamics of TTR under each buffer condition. To visualize CCS_avg_, the intensities of the CCS peaks across all observed charge states were summated to obtain a cumulative fit for each buffer (Figure S3). The CCS_avg_ of wtTTR are 3705 Å^2^, 3635 Å^2^, and 3588 Å^2^, in AmAc, EDDA, and TEAA, respectively (Figure 2A). This same trend in CCS_avg_ was observed for all TTR mutants, with CCS_avg_ consistently decreasing in EDDA and TEAA relative to AmAc (Figure 2B). The full width at half-maximum (fwhm) values for each individual charge state and buffer are reported in Supporting Information (Table S1).

**Figure 2:**
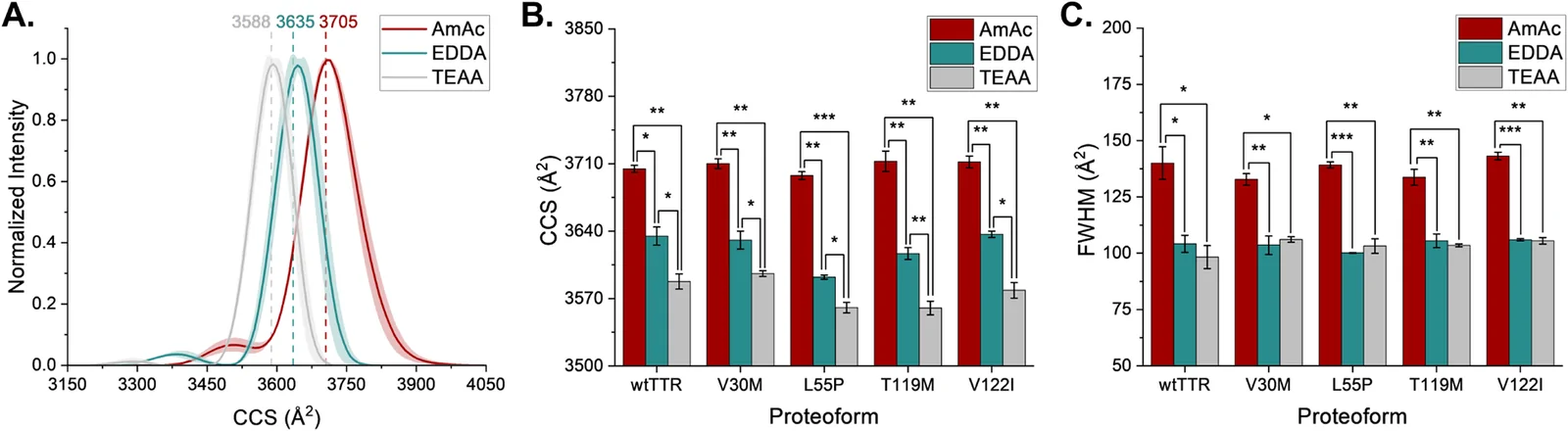
Cumulative fit of the CCS for wtTTR in AmAc, EDDA, and TEAA. **(A.)** fwhm of the larger conformers are 140 Å^2^ in AmAc, 104 Å^2^ in EDDA, and 98.3 Å^2^ in TEAA. The dotted line represents the calculated CCS_avg_ and the shaded regions indicate standard deviation between trials (*n* = 3). **(B.)** CCS_avg_ of wtTTR, V30M, L55P, T119M, and V122I, with the largest CCS_avg_ observed in AmAc, followed by EDDA, then TEAA. **(C.)** fwhm values of the most abundant conformer of wtTTR, V30M, L55P, T119M, and V122I in AmAc, EDDA, and TEAA. All proteoforms have the largest fwhm in AmAc and similar fwhm values in EDDA and TEAA. Statistical significance is indicated as *p < 0.05, **p < 0.01, ***p < 0.001 (paired two-sample *t*-test, *n* = 3). All data were collected in H_2_O.

While CCS_avg_ reflects the overall size of the protein, the range in CCS of TTR across charge states along with the fwhm of the cumulative fit provides additional insight into TTR conformational dynamics. The CCS of the most abundant wtTTR conformer in AmAc ranges from 3790 Å^2^ for the 17+ charge state to 3638 Å^2^ for the 13+ charge state, resulting in a difference of 152 Å^2^. In EDDA, this range decreases to 61 Å^2^, and to 41 Å^2^ in TEAA (Figure S4). The progressive narrowing of the CCS range indicates that wtTTR is more dynamic (greater conformational entropy) in AmAc than in EDDA or TEAA. This same trend in CCS was observed across all mutants (Figures S5–S8). Further supporting this observation is the fwhm of the cumulative fits for the most abundant conformer of wtTTR (Figure 2A). In AmAc, wtTTR has a fwhm of 140 Å^2^ in AmAc, 104 Å^2^ in EDDA, and 98.3 Å^2^ in TEAA (Figure 2C). A larger fwhm value is associated with greater conformational heterogeneity,^71^ indicating that wtTTR in AmAc has the most conformational flexibility. Alternatively, the presence of EDDA and TEAA restricts the dynamics of wtTTR relative to AmAc. Consistent with these observations, the charge state distribution of wtTTR decreases across the three buffers, with 5 charge states in AmAc, 4 in EDDA, and 3 in TEAA. This is also reflected in the Z_avg_ of wtTTR with values of 14.9 in AmAc, 12.7 in EDDA, and 11.1 in TEAA (Figure 3A), consistent with progressively more compact, less dynamic conformations exhibiting smaller SASA.^21,22^ Collectively, the CCS_avg_, fwhm, and charge-state distributions reveal that not only do EDDA and TEAA compact the overall structure of TTR, but they also hinder the dynamics of TTR (Figure 2, B–C). The opposite could also be said: TTR is the most dynamic in AmAc compared to EDDA and TEAA.

**Figure 3:**
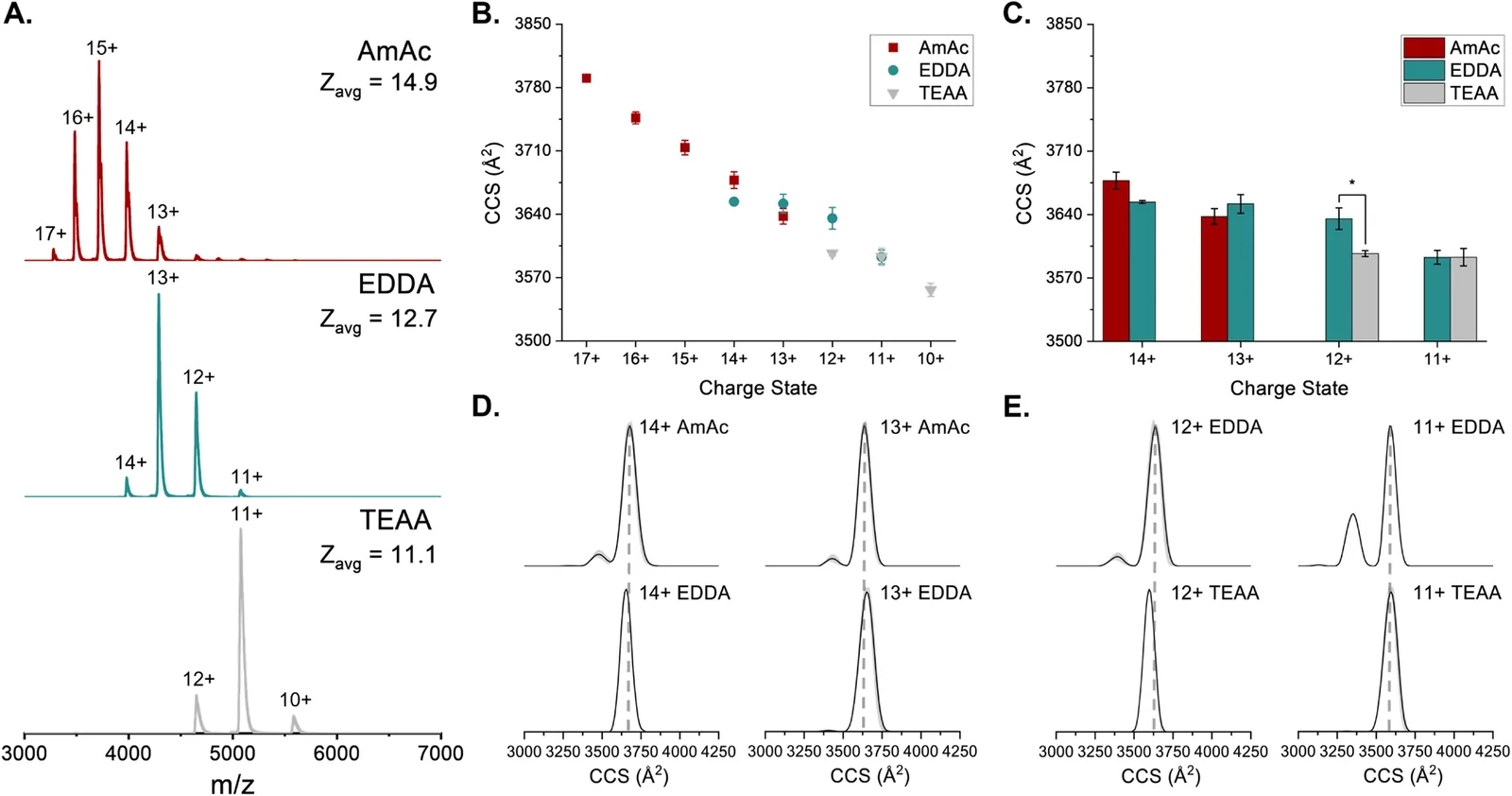
**(A.)** Native mass spectra and Z_avg_ of wtTTR in AmAc, EDDA, and TEAA. **(B.)** Scatter plot for the CCS of the most abundant conformer as a function of charge state for wtTTR in each buffer. **(C.)** Bar graph of the CCS for the most abundant conformer of wtTTR in mutual charge states. Statistical significance as indicated: *p < 0.05 (paired two-sample *t*-test, *n* = 3). **(D.)** CCS profiles of wtTTR for the 14+ and 13+ charge states of AmAc and EDDA, along with **(E.)** the 12+ and 11+ charge states of wtTTR in EDDA and TEAA; shaded regions indicate standard deviation. Reported are the mean and standard deviation (*n* = 3). Gray dashed lines are placed to better compare CCS profiles from the top profile to the bottom profile. All data were collected with the buffers in H_2_O.

A plausible explanation for these CCS_avg_ shifts could be the differences in the inherent properties of the buffers themselves. At pH 7.0, the EDDA cation has two protonated amine groups capable of interacting with multiple sites on the protein, potentially serving as a noncovalent kinetic trap or cross-linker, and restricting TTR dynamics. The TEAA cation is a hydrophobic compound capable of interacting with different sites of TTR, thereby perturbing the hydration differently compared to the more hydrophilic cations of AmAc or EDDA,^72^ resulting in the more compact structure seen in Figure 2A. Similar observations were seen across all proteoforms of TTR tested, further supporting the claim that buffer molecules alter the physicochemical properties of TTR tetramers (Figure S9).

Some seemingly contradictory data are that TTR in TEAA shows %D levels comparable to those in AmAc (Figure 1), despite having a significantly smaller CCS_avg_ (Figure 2B). This relates to what HDX data report and the interpretation thereof. HDX data report on regions of the protein that are available for exchange with the surrounding solvent (i.e., SASA)^12,70^ but might not directly relate to a more extended or compact protein structure. It is also important to remember that intact protein HDX-MS only reports on how many hydrogen atoms are exchanging with deuterium and not on where the exchange occurs. Additionally, with intact protein HDX-MS, the uptake of deuterium also occurs on the side chains of amino acid residues and not just on the backbone. Thus, although IM-MS data reveal that TEAA compacts the structure and restricts the dynamics of TTR, the overall solvent accessibility of the regions for exchange remains similar to that in AmAc. Because TEAA is more hydrophobic (Table 1), it may interact with hydrophobic residues more than hydrophilic residues, leaving more hydrophilic residues exposed whose side chains can undergo HDX, resulting in a similar %D to AmAc. Alternatively, TEAA could disrupt hydrogen bonds or salt bridges that exist in the presence of AmAc, thereby increasing local D_2_O accessibility to hydrogen atoms on the residues despite global compaction. In either case, similar %D values for intact protein HDX experiments do not directly indicate similar structures but rather indicate comparable solvent accessibilities.

### IM-MS Reveals Different CCS Conformers across Mutual Charge States

Charge states in nMS can provide information regarding protein dynamics, stability, and structure.^22,24,73,74^ For this reason, the CCS of TTR was compared across mutual charge states shared between AmAc and EDDA, and between EDDA and TEAA. Two charge states of wtTTR, 14+ and 13+, are seen in both AmAc and EDDA, whereas EDDA and TEAA have charge states 12+ and 11+ in common (Figure 3A). No significant difference in CCS for the most abundant conformer of the 14+ (3678 Å^2^, 3654 Å^2^) and 13+ (3638 Å^2^, 3652 Å^2^) charge states in AmAc and EDDA was observed. Similarly, no significant difference was observed for the 11+ charge state between EDDA and TEAA (3593 Å^2^, 3593 Å^2^). The 12+ charge state of wtTTR in EDDA and TEAA did show a significant difference in CCS for the most abundant conformer (3635 Å^2^ and 3597 Å^2^, respectively) (Figure 3C).

Although the CCS values of the most abundant conformers show limited significance across shared charge states, differences can be seen in the CCS profiles. The 14+ and 13+ charge states of wtTTR have two conformers present in AmAc, whereas only one conformer is visible in EDDA (Figure 3D). The same can be said for EDDA and TEAA, where the 12+ and 11+ charge states of wtTTR exhibit two conformers in EDDA but only one in TEAA (Figure 3E). These patterns were consistent across all other proteoforms of TTR tested (Figures S10–S13).

Overall, these data indicate that although the CCS values may be similar across mutual charge states, the conformational populations differ across the buffers. This likely reflects how buffer-specific effects shift the hydration and structural landscape of TTR, leading the protein to adopt and favor different conformational populations at the same charge state. Moreover, the absence of the smaller conformer of TTR in EDDA relative to AmAc for the shared 14+ and 13+ charge states, and in TEAA relative to EDDA for the shared 12+ and 11+ charge states (Figure 3D–E), further supports the conclusion that EDDA and TEAA restrict the conformational dynamics of TTR.

### Native MS Buffers Influence Tetramer Stability

TTR forms a tetramer whose stability is very sensitive to its local environment. Tetramer instability can lead to subunit exchange (SUE), where the tetramer disassembles into monomers/dimers, which then reassemble as a tetramer.^49,75,76^ In previous studies, tracking this process required modifications to TTR, such as mass tags, substantial isotopic labeling, or mixing different proteoforms of TTR.^49,64,77–79^ One unprecedented benefit of the intact protein HDX-MS experiments performed here was the ability to directly observe tetramer disassembly and reassembly over time without significant modifications to TTR. This behavior was observed for wtTTR and was especially prominent for proteoforms L55P and V122I (Figure 4A–C).

**Figure 4:**
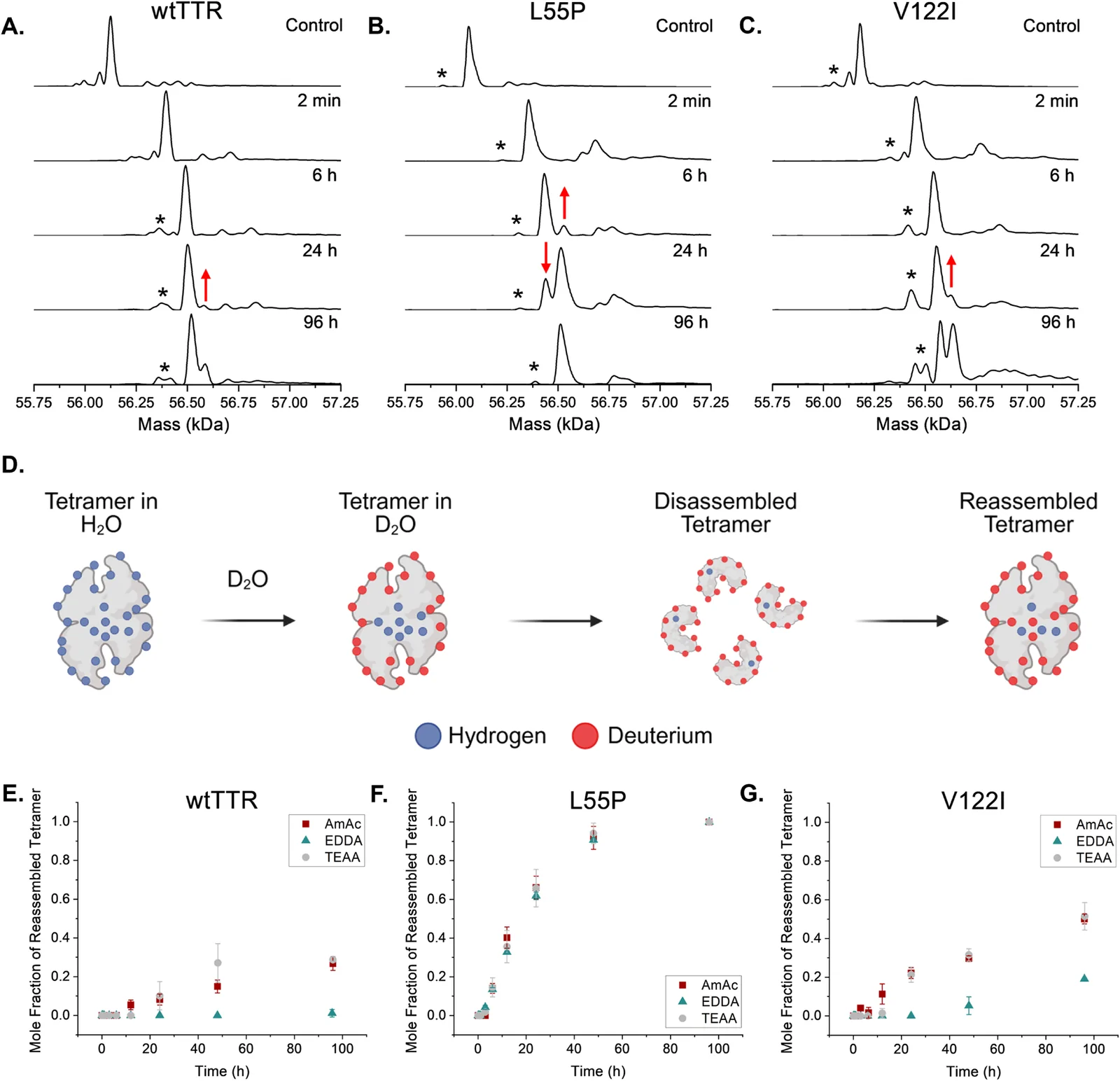
Mass shift that occurs over time after dilution of **(A.)** wtTTR, (**B.)** L55P, and **(C.)** V122I with 200 mM AmAc prepared in D_2_O. Asterisks (*) indicate 3 NEM bound to the TTR tetramer rather than 4 (most abundant peak). Red arrows indicate the emergence of a second, higher mass peak at longer time points. The abundance of the higher mass peak (reassociated tetramer) increases over time, while the abundance of the lower mass peak (original tetramer) decreases. **(D.)** Proposed mechanism to explain the appearance of the higher mass peak observed: when initially exposed to D_2_O, TTR remains in its original form, and its residues undergo HDX in the first observed peak. Over time, the tetramer dissociates into monomers, exposing residues previously blocked from the solvent, which allows for additional HDX. When the monomers reassemble into the tetramer, a second, higher mass peak appears, reflecting the increased level of deuterium incorporation. Mole fraction plot of the reassembled tetramer of **(E.)** wtTTR, **(F.)** L55P, and **(G.)** V122I as a function of time. wtTTR and V122I have varying levels of tetramer reassembly after 96 h in EDDA compared to AmAc or TEAA. In contrast, L55P undergoes complete disassembly and reassembly over the 96 h, independent of the buffer solution. Mean and standard deviation are reported (*n* = 3).

The clearest example is for L55P, where a second, higher-mass peak emerged around 3 h. This peak gradually increases in intensity, while the original, lower-mass peak decreases and eventually disappears after 96 h (Figure 4B). This second, higher-mass peak is interpreted to be a product of the disassembly and reassembly process. An explanation as to how this second, higher mass species of the TTR tetramer forms is provided in Figure 4D. The original mass shift observed after 2 min (Figure 4A–C) is due to the initial burst of HDX that occurs upon diluting the TTR sample with D_2_O. As the TTR tetramer disassembles into monomers over time, residues previously buried within the quaternary structure of TTR are now exposed to D_2_O. These newly exposed residues on the monomers exchange with the deuterium, thereby increasing the overall mass as the monomers reassemble into the tetramer (see Figures S14–S16).

To confirm that reassembly occurred, an additional experiment was performed. One apo-L55P sample was mixed with a different L55P sample where *N*-ethylmaleimide (NEM) was covalently bound to the 1 cysteine residue on each subunit. Initially, only the 4 NEM- and 0 NEM-bound L55P were observed. However, over time, intermediates with 1, 2, and 3 NEM-bound species appeared (Figure S17). This experiment validates that L55P undergoes the disassembly/reassembly process and that the higher-mass peak in the intact protein HDX experiment represents the reassembled tetramer.

To determine how SUE was affected by the buffers present, the mole fraction of reassembled tetramer vs the original tetramer was calculated (see Methods). L55P began this disassembly/reassembly process after ∼3 h, and by 96 h, all TTR present in solution had reassembled across all three buffers (Figure 4F).

V122I has a more stable tetramer than L55P, as the disassembly/reassembly process does not occur completely after 96 h. Its reassembled tetramer mole fractions at 96 h were 0.50 in AmAc, 0.51 in TEAA, and 0.19 in EDDA (Figure 4G). Wild-type TTR demonstrates even greater tetramer stability with reassembled mole fractions of 0.27 in AmAc, 0.29 in TEAA, and 0.01 in EDDA at 96 h (Figure 4E). It would be expected to see the reassembled product appear in the experiments with V30M due to its known tetramer instability,^49^ however, Na adducts made it difficult to distinguish adducts from reassembled tetramer products (Figure S18). No evidence of reassembly was observed for T119M (Figure S19). A previous study that reported bottom-up HDX data on various TTR tetramers determined that hydration at the dimer–dimer interface dictates complex stability.^23^ The data in this present study determine that SUE behavior is altered depending on the buffer molecules present, suggesting that buffer molecules alter hydration at the TTR interface, shifting tetramer stability.

One notable observation is that the disassembly/reassembly rate of L55P was unaffected by the buffer identity, with similar reassembled tetramer mole fraction values across all three buffers. In contrast, wtTTR and V122I displayed clear buffer-dependent behavior for EDDA. Although reassembly of both mutants started slightly earlier in AmAc than in TEAA, similar mole fraction values were observed in the two buffers after 96 h (Figure 4E,G). In EDDA, V122I did not begin to reassemble until 48 h and showed significantly less reassembly by 96 h compared with AmAc or TEAA (Figure 4G). For wtTTR, reassembly in EDDA was observed in only one replicate (Figure S1B). These results indicate that EDDA stabilizes the tetramers of both V122I and wtTTR, slowing down the disassembly and reassembly rate (Figure 4E,G). This could be due to the previously reported kosmotropic behavior of EDDA, where EDDA may promote more structure within the hydration shell,^53^ therefore stabilizing the tetramers.

Tetramer instability has long been associated with ATTR, and both V122I and L55P are more aggregation-prone than wtTTR.^80–82^ This increased instability is perceived in the rate at which the L55P and V122I tetramers disassemble/reassemble compared to wtTTR (Figure 4E–G). The water network, or hydration, at the dimer–dimer interface of TTR is known to stabilize the tetramer,^49,83^ and Lashuel et al. showed that L55P has a lower activation energy barrier for tetramer disassembly than wtTTR.^84^ This suggests that the L55P mutation disrupts the stabilizing water network such that any additional stabilization provided by EDDA for wtTTR or V122I is ineffective for L55P. Although wtTTR and V122I began to disassemble/reassemble around the same time, V122I has a larger population of reassembled tetramers than wtTTR at 96 h. wtTTR appears to receive more of a stabilization effect from EDDA than V122I, another indication that the V122I tetramer is less stable than the wtTTR tetramer. T119M stands apart in that no disassembly or reassembly was detected (Figures S1J–L and S18), consistent with its well-established tetramer stability and known protective role against amyloidosis.^85–87^

### TTR Mutants Alter Tetramer Hydration

While varying buffer conditions is one way to alter TTR hydration, missense mutations are another way to do so.^50^ In the intact protein HDX-MS experiments, V30M showed significantly higher %D uptake than wtTTR in both AmAc and EDDA (Figure 5A). In contrast, IM-MS data revealed that V30M has a CCS_avg_ comparable to that of wtTTR (Figure 5B). Together, these experiments suggest that although the overall CCS of V30M is similar to that of wtTTR, this mutation increases solvent accessibility and disrupts the water network surrounding TTR. This shift in hydration could additionally influence the protein behavior and dynamics.

**Figure 5:**
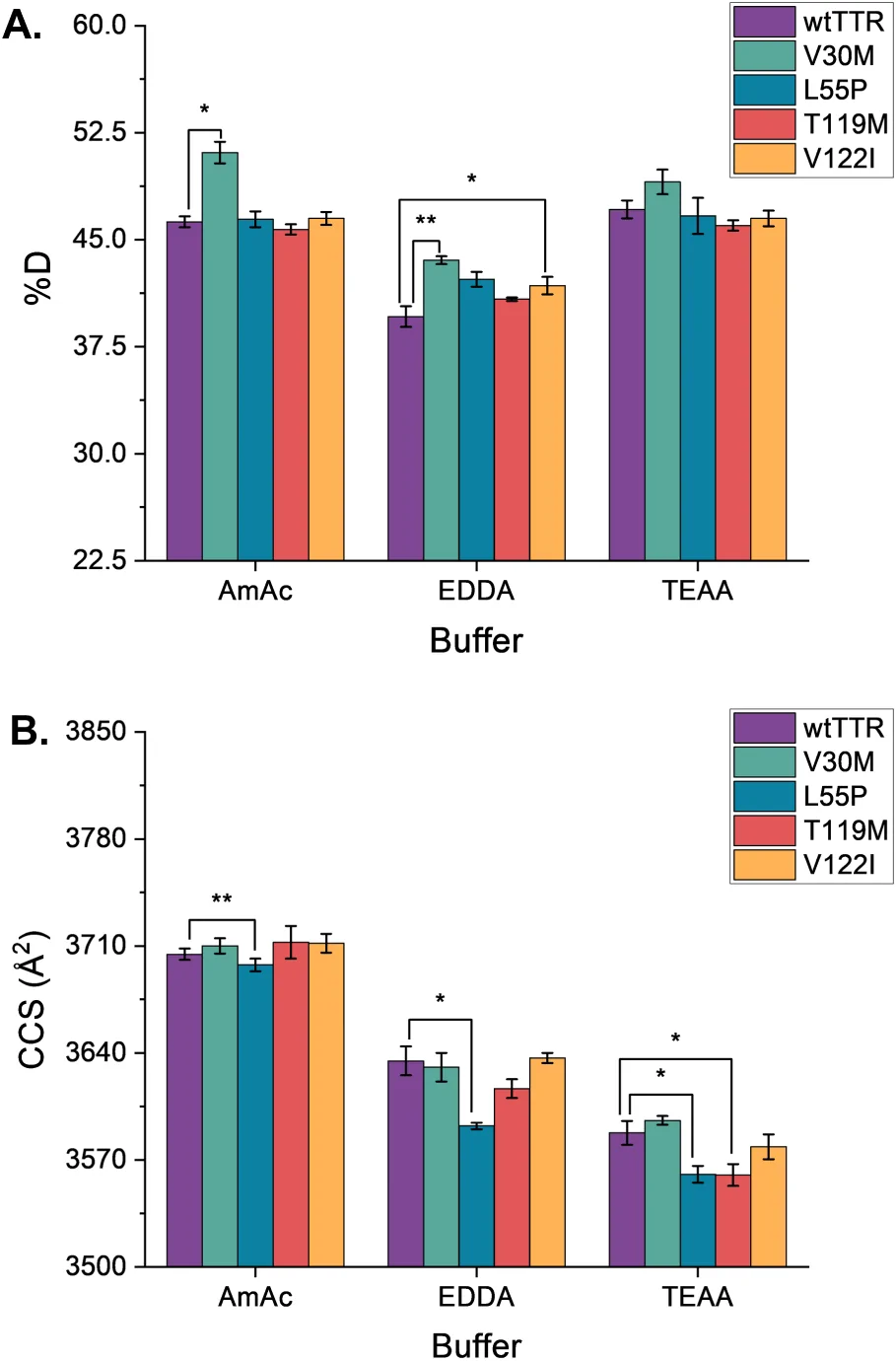
**(A.)** Percent deuterium of the original tetramer of wtTTR, V30M, L55P, T119M, and V122I at 48 h. **(B.)** CCS_avg_ of all five TTR proteoforms (wtTTR, V30M, L55P, T119M, V122I) in AmAc, EDDA, and TEAA in H_2_O. Reported are the mean and standard deviation (*n* = 3). Statistical significance is indicated as *p < 0.05, **p < 0.01 (paired two-sample *t*-test) and was only compared against wtTTR.

L55P shows opposite behavior to V30M, having a significantly smaller CCS_avg_ than wtTTR in all three buffers yet similar %D. Despite this more compact structure, L55P may be losing stabilizing intramolecular bonds that would limit exchange, resulting in a %D that is similar between L55P and wtTTR. The L55P mutation could also be perturbing the stabilizing water molecules at the dimer–dimer interface, resulting in a more compact yet less stable tetramer. This aligns with previous literature, where L55P was reported as highly unstable and theorized to disrupt hydration.^23,61,84^

Interestingly, many more similarities than differences are seen for %D and CCS_avg_ between proteoforms in the same buffer. A similar issue arises when comparing crystal structures of TTR and TTR mutants: they all are comparable to each other, making it difficult to predict what exactly induces tetramer instability and further aggregation.^88^ Shifts in hydration could be one explanation for these observations. If a mutation disrupts stabilizing water networks or alters the way that water interacts with certain regions of the protein, those differences may be too small to notice in a crystal lattice, but they can be determined by altering solution chemistry and analyzing the products using nMS methods. For instance, the statistical significance of the data obtained is inconsistent for the different TTR proteoforms between buffers (Tables S2 and S3). V122I differs significantly from wtTTR in %D only in EDDA, while T119M differs significantly in CCS_avg_ compared to wtTTR only in TEAA. This demonstrates how AmAc, EDDA, and TEAA can affect protein hydration differently and sheds light on differences between proteins not typically seen when using one-buffer systems.

## Conclusions

The results described in this work reveal that commonly used nMS buffers — AmAc, EDDA, and TEAA — influence a protein’s physicochemical properties via noncovalent interactions with the protein, altering the surrounding hydration shell. Solutions containing EDDA decrease the deuterium uptake of all TTR proteoforms relative to AmAc or TEAA solutions. This decrease in uptake is evidence that EDDA has a stronger interaction with TTR compared to AmAc or TEAA, therefore reducing the SASA and suppressing HDX. It is also possible that the additional amine group on the cation of EDDA interacts with multiple residues on the protein simultaneously, acting as a kinetic trap. Recent studies have shown how both TEAA and EDDA alter ligand binding compared to AmAc on GroEL, which was attributed to different interactions of the buffer molecules with the protein.^53^ These studies support our conclusion that the %D of TTR is inhibited due to noncovalent interactions between EDDA and TTR that occur in solution.

Noncovalent interactions involving buffer binding to the protein alter protein hydration by restricting access of the solvent to the protein, altering the SASA. Shifts in hydration of a protein complex have been shown to alter the stability and dynamics of protein complexes, including TTR.^23,42,49–51^ In AmAc solutions, TTR has the largest CCS_avg_ and fwhm, e.g., conformational entropy, whereas EDDA and TEAA solutions restrict the dynamics and conformational entropy. In addition, the presence of EDDA reduces tetramer disassembly and reassembly for wtTTR and V122I, revealing that buffers can also influence protein stability. EDDA may stabilize TTR by restructuring the surrounding hydration to be more ordered, therefore inhibiting the dynamics of TTR and acting as a form of a kinetic trap. It is known that salts, or buffer molecules, can alter protein hydration shells;^40,41,52^ we interpret differences in IM-MS and intact protein HDX-MS results as evidence that the changes seen in the physicochemical properties of TTR in the different buffers occur by noncovalent interactions that alter hydration.

The results shown here underscore the importance of considering how buffer contents may alter protein behavior and results of biochemical assays. Previous studies reported by Oney-Hawthorne et al. demonstrated that both nMS and traditional biological buffers altered the enzymatic activity of the cysteine desulfurase, IscS.^6^ Richter et al. showed that clerocidin, an inhibitor of topoisomerase II, covalently interacts with TRIS, a commonly used biological buffer, significantly decreasing its potency.^89^ Additionally, Salis and Monduzzi report additional examples of biological buffers influencing enzyme activity, protein electrophoretic mobility, and aggregation.^90^ Understanding the role of buffers in shaping the physicochemical properties of proteins in solution is essential for experiments involving proteins and protein complexes. This includes, but is not limited to, drug formulation, enzyme activity, protein–ligand binding, and protein aggregation studies.

## Supplementary Material



## Notes

The mass shifts resulting from the addition of D_2_O for intact protein HDX experiments, intact protein HDX-MS plots for all trials across all proteoforms for the three buffers, additional control experiments, IM-MS data, and data tables for both intact protein HDX-MS and IM-MS experiments (PDF)
