# Supplementary material for: Solution Phase Protein Dynamics: Influences of ESI Buffers on Hydration
Source: Anal Chem. 2026 Aug 10;98(33):24155–66. doi: 10.1021/acs.analchem.6c02162 (PMC13523555; doi:10.1021/acs.analchem.6c02162)
Supplement: Supplementary file 1 [file ac6c02162_si_001.pdf]

Supplemental Information

Solution Phase Protein Dynamics: Influences of ESI Buffers on Hydration

Emily Burningham, Carter Lantz, Robert L. Rider, Syuan-Ting Kuo, Sangho D. Yun, Arthur Laganowsky, David H. Russell*

Department of Chemistry, Texas A&M University, College Station, Texas 77843

*Corresponding author; email: russell@chem.tamu.edu

Table of Contents

Figure S1: %D across all proteoforms and buffers S2
Figure S2: %D of wtTTR comparing 200 mM AmAc vs 67 & 200 mM EDDA S3
Figure S3: CCS cumulative fit of wtTTR in AmAc, EDDA, and TEAA S3
Figure S4: CCS profile of wtTTR in Amac, EDDA, and TEAA S4
Figure S5: CCS profile of V30M in Amac, EDDA, and TEAA S4
Figure S6: CCS profile of L55P in Amac, EDDA, and TEAA S5
Figure S7: CCS profile of T119M in Amac, EDDA, and TEAA S5
Figure S8: CCS profile of V122I in Amac, EDDA, and TEAA S6
Figure S9: CCS cumulative fit of V30M, L55P, T119M, V122I in all buffers S7
Figure S10: CCS and conformers of L55P across mutual charge states S8
Figure S11: CCS and conformers of L55P across mutual charge states S9
Figure S12: CCS and conformers of T119M across mutual charge states S10
Figure S13: CCS and conformers of V122I across mutual charge states S11
Figure S14: wtTTR HDX mass shift S12
Figure S15: L55P HDX mass shift S12
Figure S16: V122I HDX mass shift S13
Figure S17: SUE of L55P with NEM S13
Figure S18: V30M HDX mass shift S14
Figure S19: T119M HDX mass shift S14
Table S1: FWHM (CCS) for every proteoform, charge state, and buffer S15
Table S2: *p*-values across all proteoforms and mutants - HDX S16
Table S3: *p*-values across all proteoforms and buffers - CCS_{avg} S16

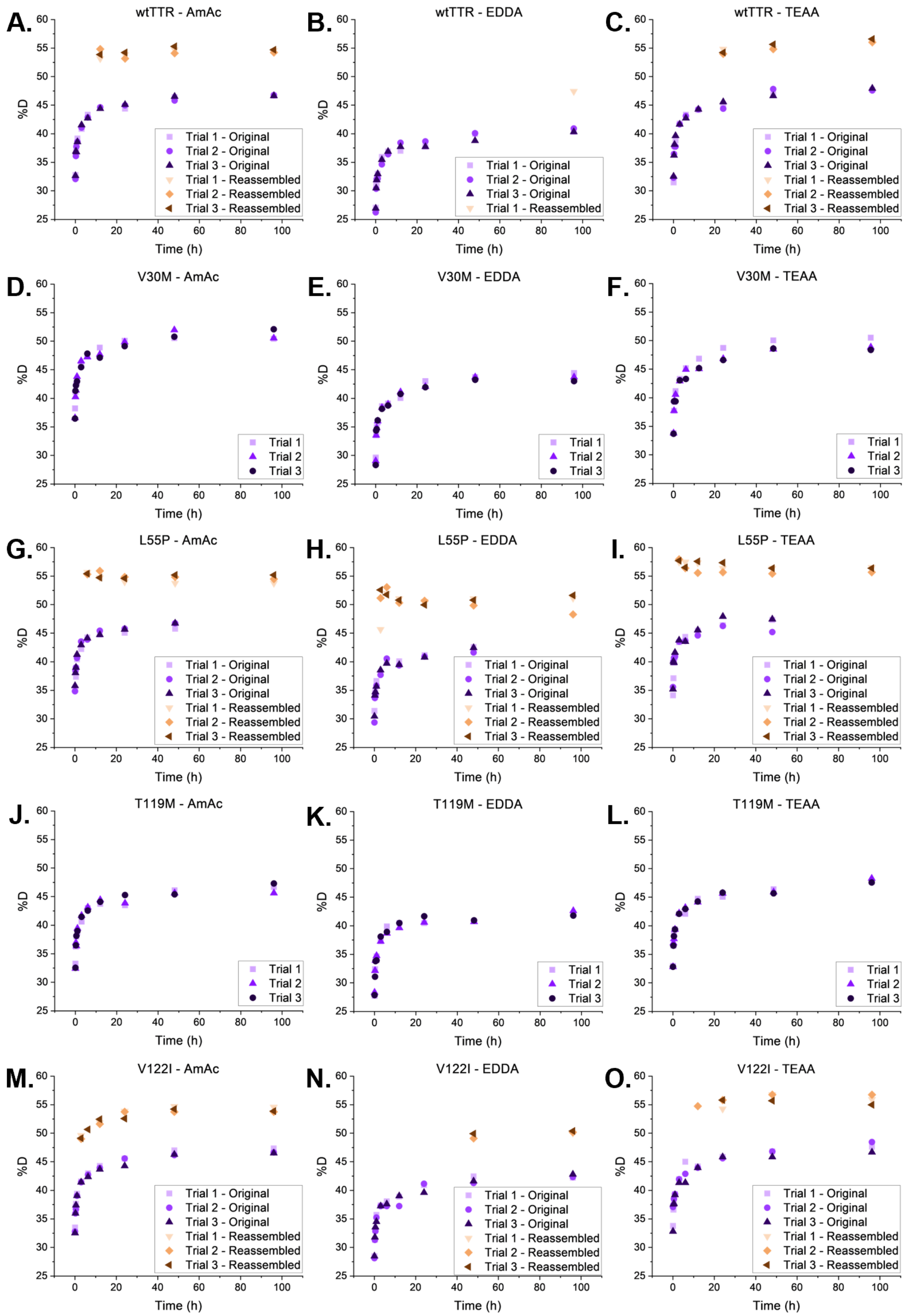

Figure S1: HDX data from three trials in each buffer (AmAc, EDDA, and TEAA) for wtTTR **(A-C)**, V30M **(D-F)**, L55P **(G-I)**, T119M **(J-L)**, and V122I **(M-O)** in. For wtTTR in EDDA **(B.)** only one trial indicated the presence of the reassembled tetramer.

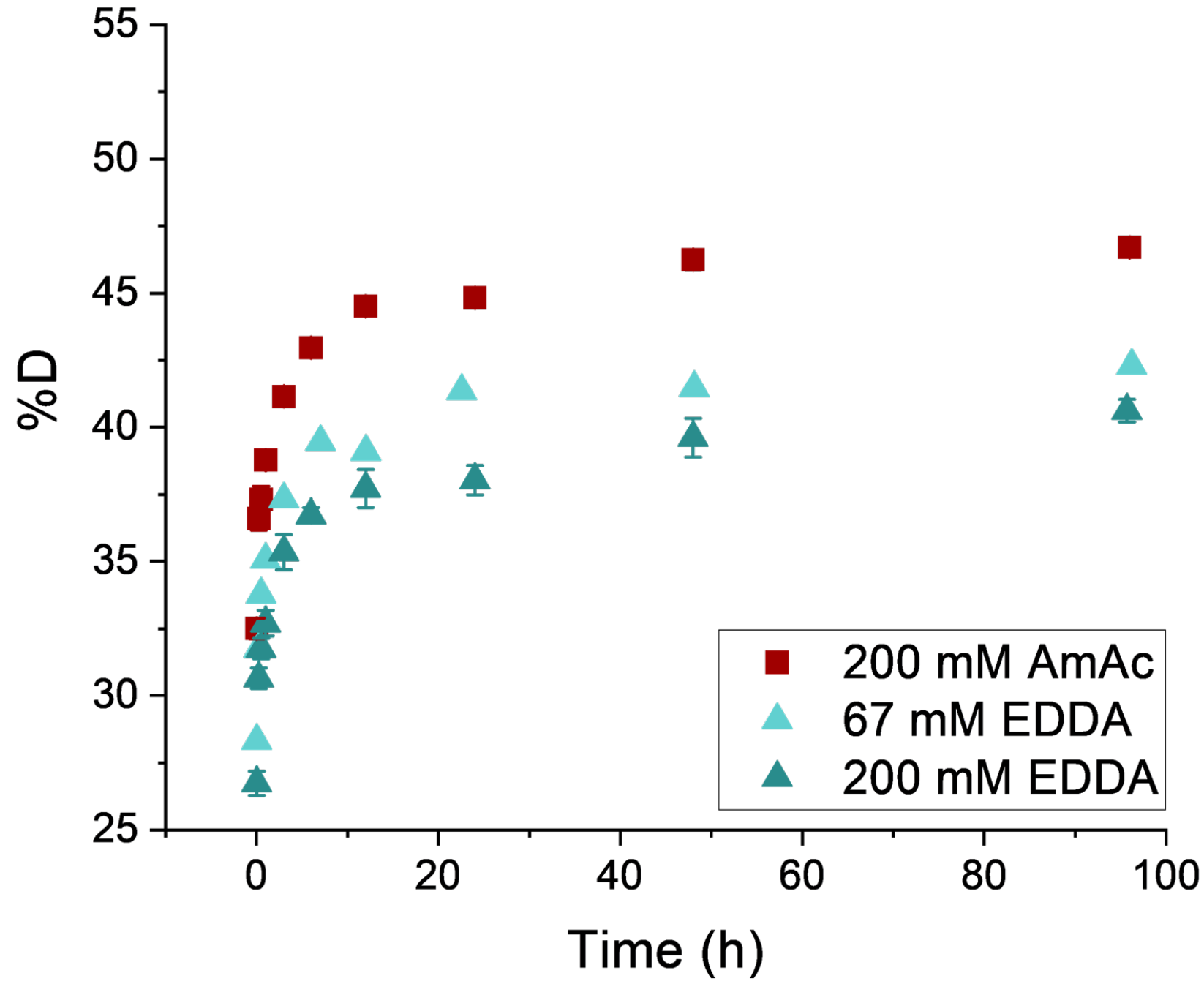

Figure S2: HDX of wtTTR in 200 mM AmAc (3 trials), 67 mM EDDA (1 trial), and 200 mM EDDA (3 trials).

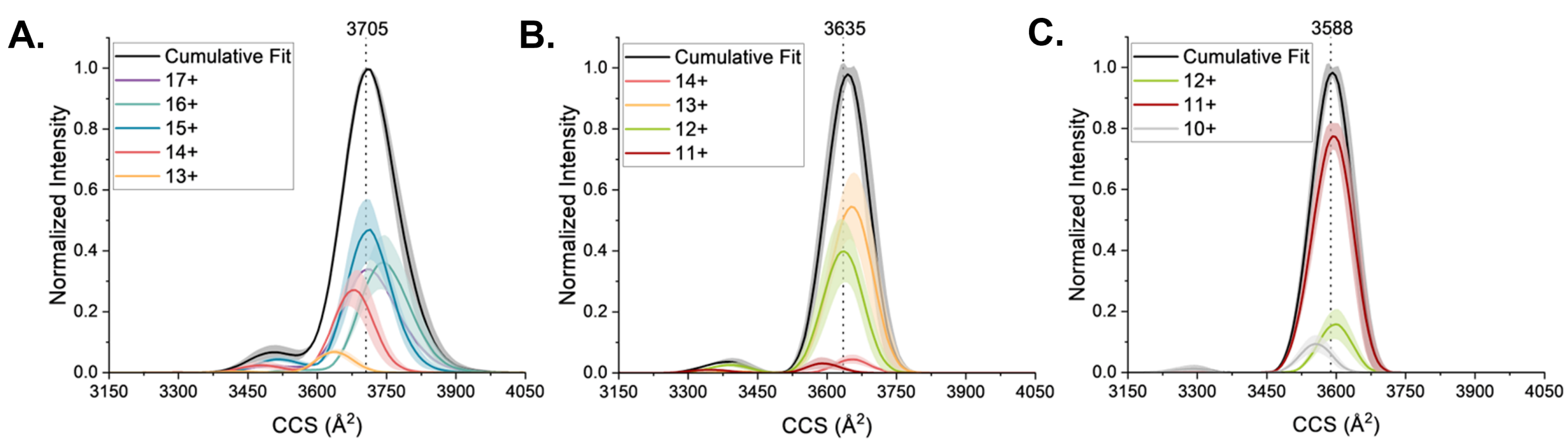

Figure S3: CCS_{avg} of wtTTR in AmAc **(A.)**, EDDA **(B.)**, and TEAA **(C.)**. The dotted line represents the CCS_{avg} determined where the black line represents the cumulative fit of the CCS for all charge states combined. The shaded region indicates standard deviation between trials (n = 3).

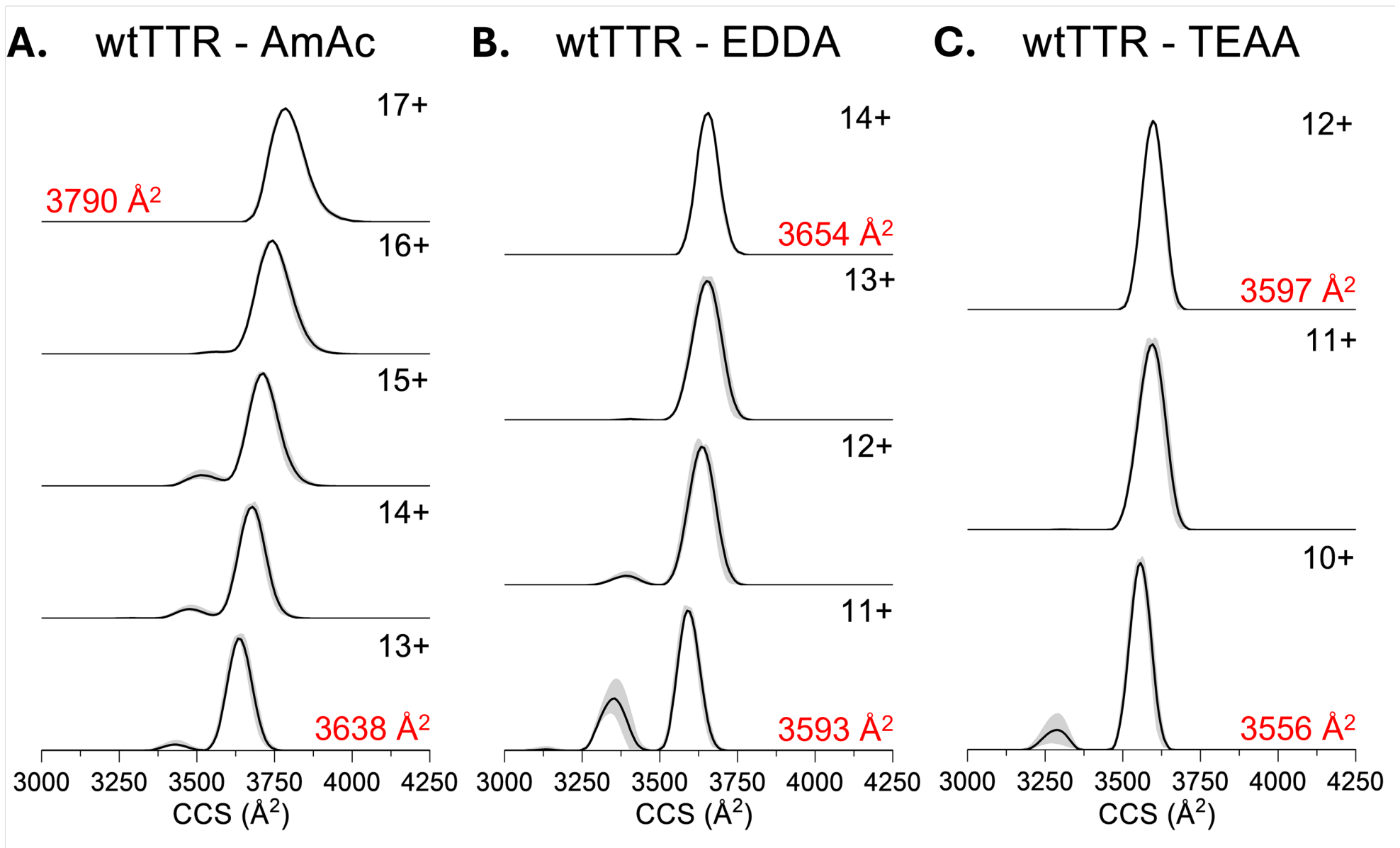

Figure S4: CCS profile of wtTTR in AmAc **(A.)** EDDA **(B.)** and TEAA **(C.)**. Reported are mean and standard deviation (n = 3) where the shaded region indicates standard deviation. CCS of the most abundant conformer for highest and lowest charge states of all buffers shown in red. Range of these CCS values are 152 Å^2, 61 Å^2, and 41 Å^2 for AmAc, EDDA, and TEAA respectively.

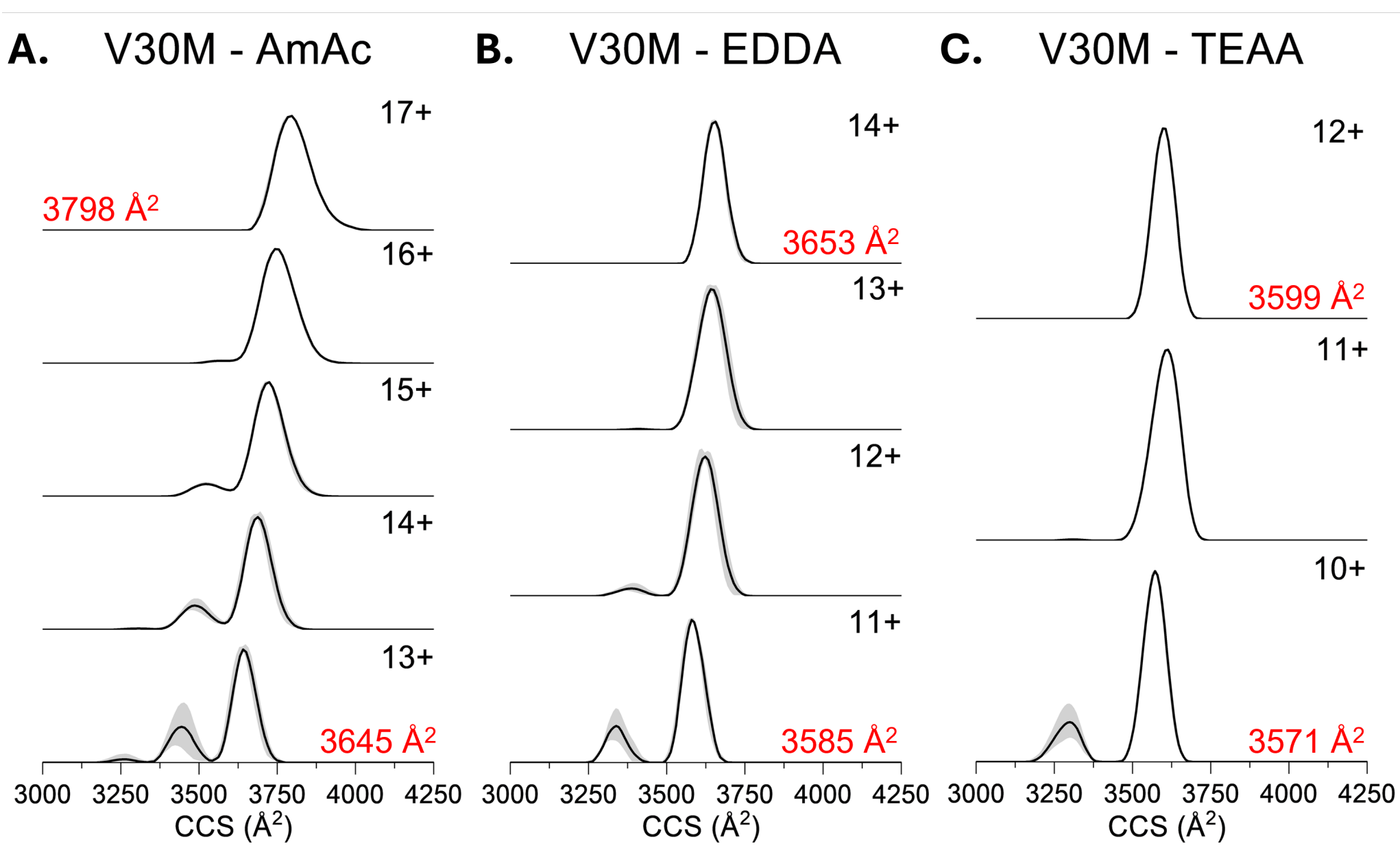

Figure S5: CCS profile of V30M in AmAc **(A.),** EDDA **(B.),** and TEAA **(C.)**. Reported are mean and standard deviation (n = 3) where the shaded region indicates standard deviation. CCS of the most abundant conformer for highest and lowest charge states of all buffers shown in red. Range of these CCS values are 153 Å^2, 68 Å^2, and 28 Å^2 for AmAc, EDDA, and TEAA respectively.

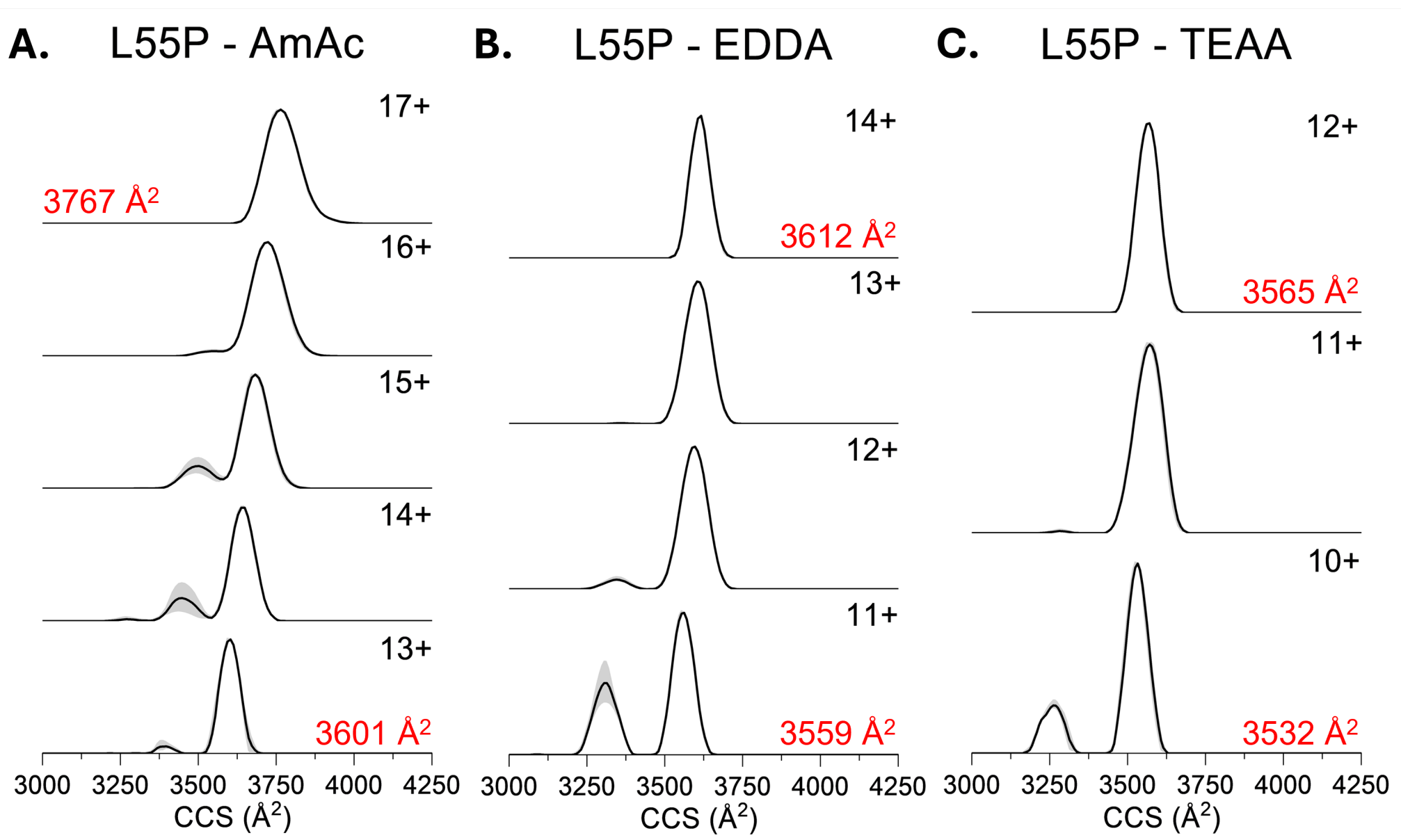

Figure S6: CCS profile of L55P in AmAc **(A.)**, EDDA **(B.)**, and TEAA **(C.)**. Reported are mean and standard deviation (n = 3) where the shaded region indicates standard deviation. CCS of the most abundant conformer for highest and lowest charge states of all buffers shown in red. Range of these CCS values are 166 Å^2, 53 Å^2, and 33 Å^2 for AmAc, EDDA, and TEAA respectively.

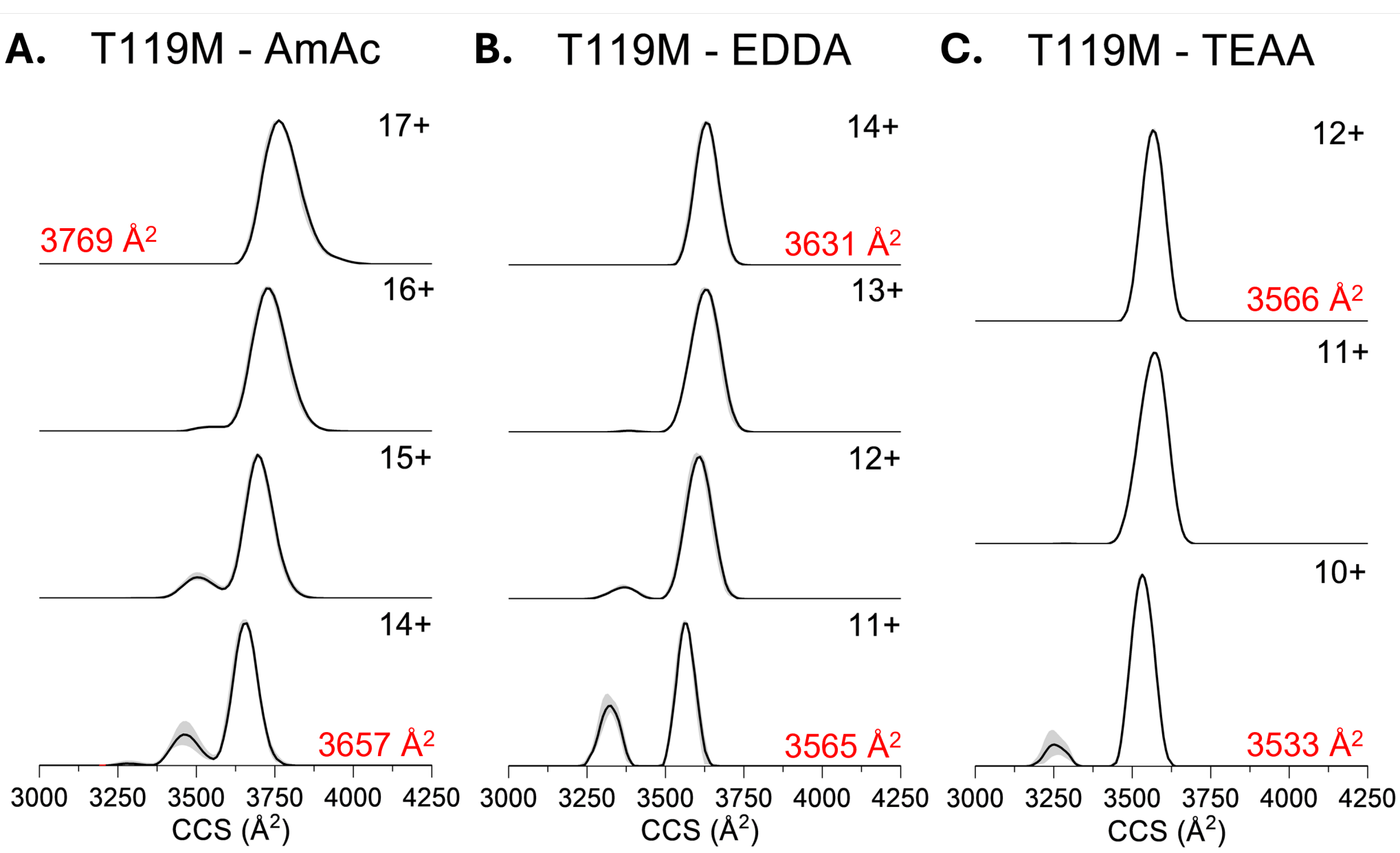

Figure S7: CCS profile of T119M in AmAc **(A.)**, EDDA **(B.)**, and TEAA **(C.)**. Reported are mean and standard deviation (n = 3) where the shaded region indicates standard deviation. CCS of the most abundant conformer for highest and lowest charge states of all buffers shown in red. Range of these CCS values are 166 Å^2, 53 Å^2, and 33 Å^2 for AmAc, EDDA, and TEAA respectively.

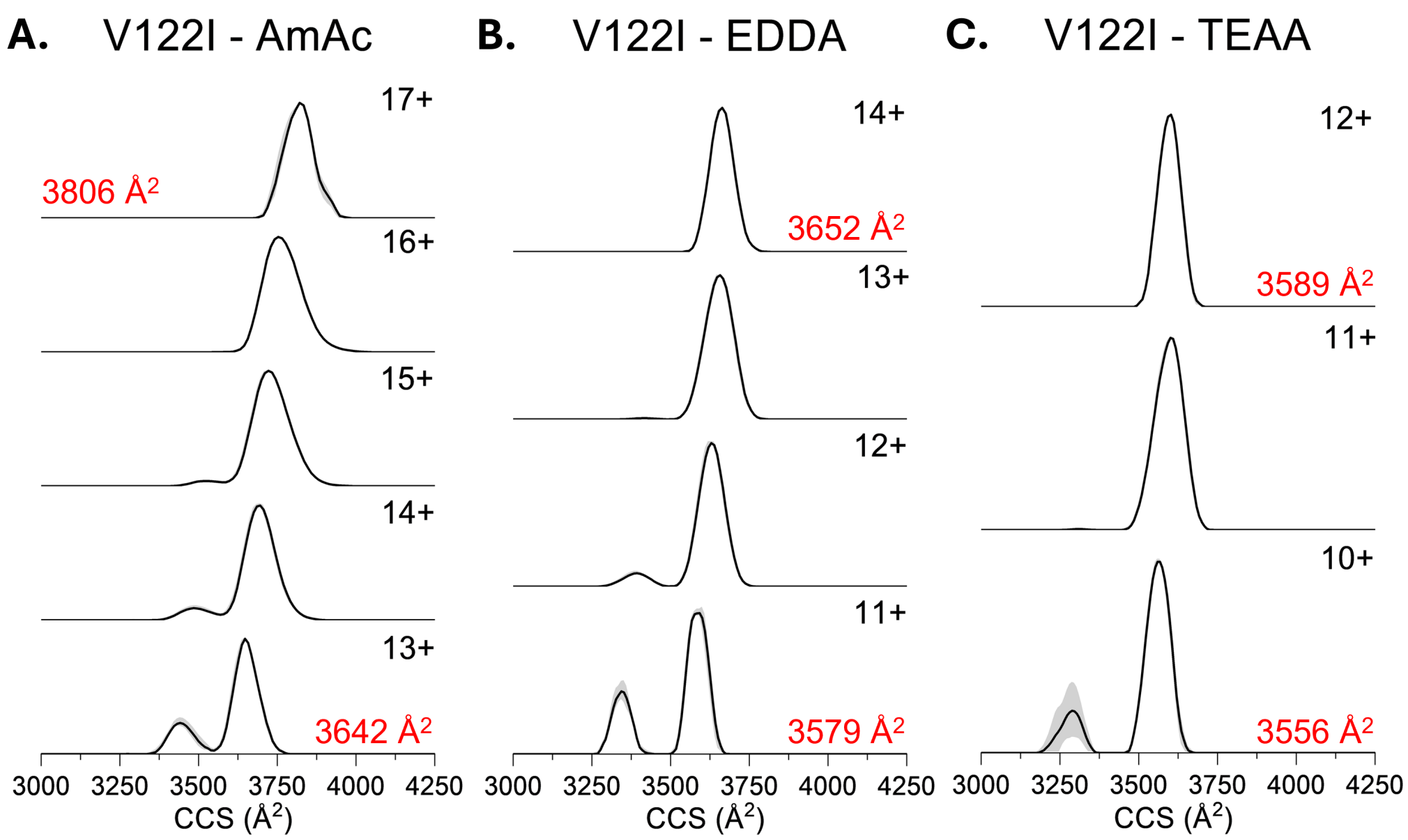

Figure S8: CCS profile of V122I in AmAc **(A.)**, EDDA **(B.)**, and TEAA **(C.)**. Reported are mean and standard deviation (n = 3) where the shaded region indicates standard deviation. CCS of the most abundant conformer for highest and lowest charge states of all buffers shown in red. Range of these CCS values are 164 Å^2, 73 Å^2, and 33 Å^2 for AmAc, EDDA, and TEAA respectively.

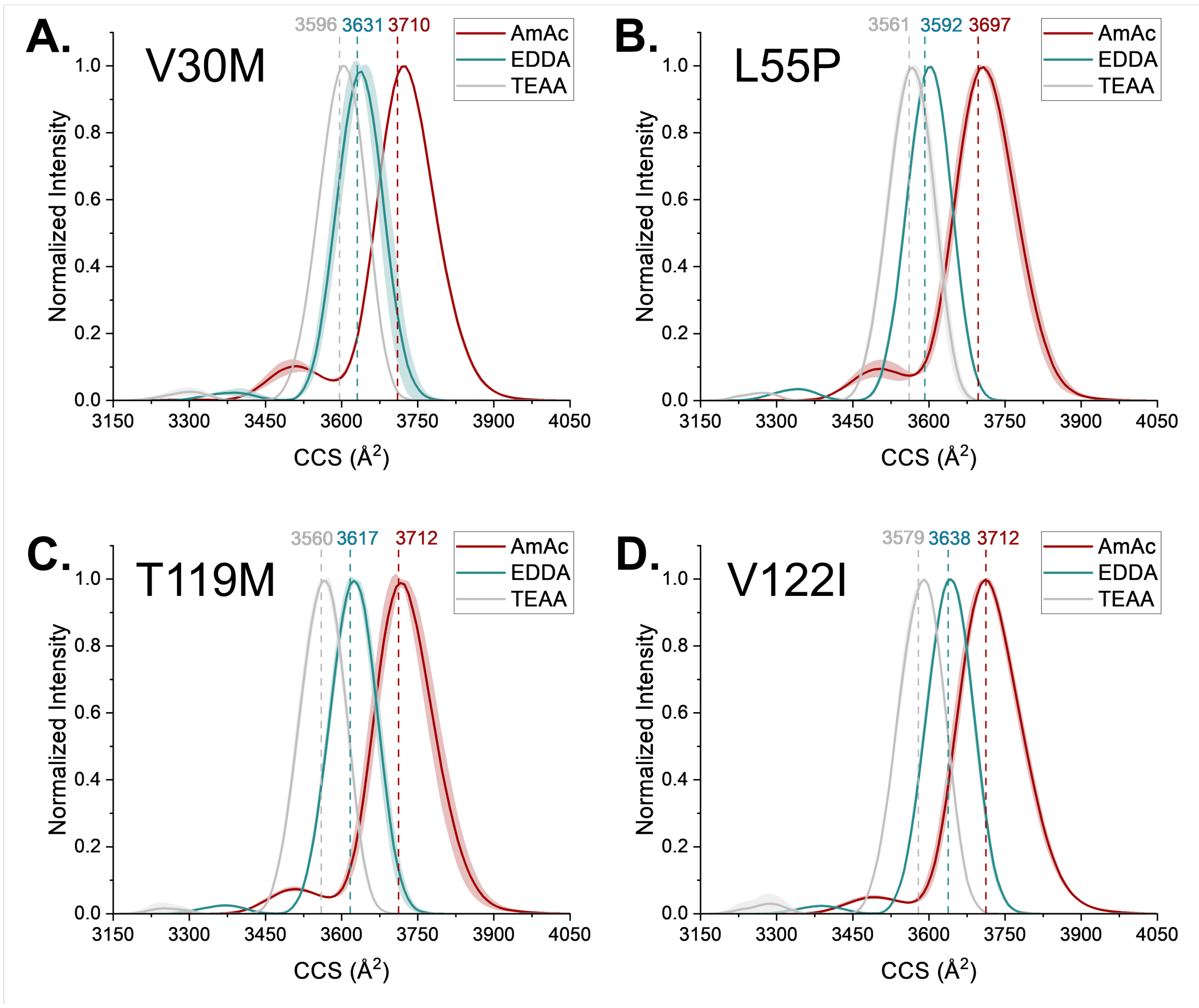

Figure S9: Cumulative fit of the CCS for V30M in AmAc, EDDA, and TEAA **(A.)** Cumulative fit of the CCS for L55P in AmAc, EDDA, and TEAA **(B.)** Cumulative fit of the CCS for T119M in AmAc, EDDA, and TEAA **(C.)** Cumulative fit of the CCS for V122I in AmAc, EDDA, and TEAA **(D.)**

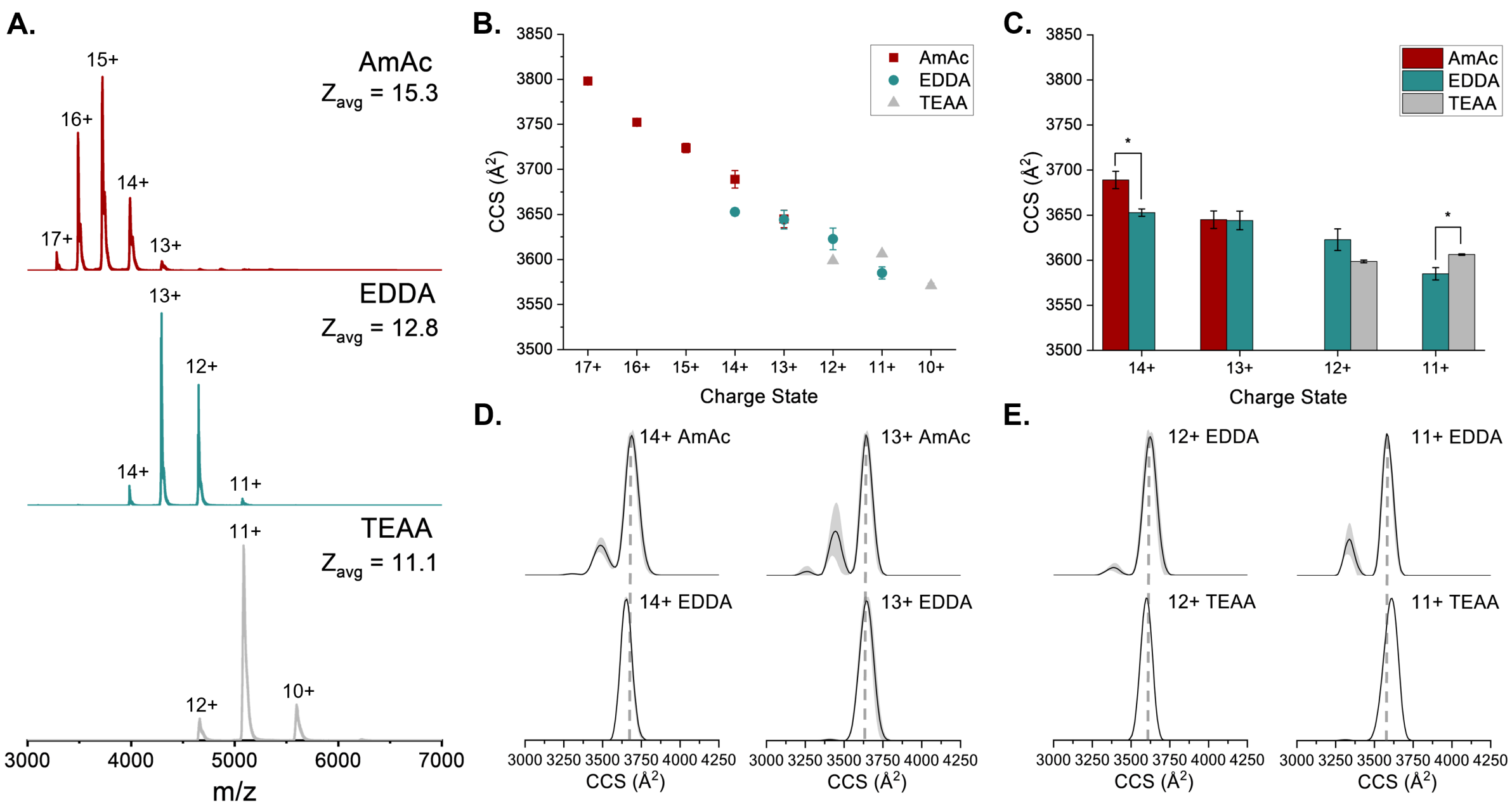

Figure S10: Native mass spectra and Z_{avg} of V30M in AmAc, EDDA, and TEAA **(A.)**. Scatter plot of the CCS of the most abundant conformer as a function of charge state for V30M in each buffer **(B.)**. Bar graph of the CCS for the most abundant conformer of V30M in overlapping charge states **(C.)**. CCS profiles of V30M for the 14+ and 13+ charge states of AmAc and EDDA **(D.)** along with the 12+ and 11+ charge states of V30M in EDDA and TEAA **(E.)**; shaded regions indicate standard deviation. Reported are the mean and standard deviation (n=3). Statistical significance as indicated: *$p < 0.05$ (paired two sample t-test, n = 3). Gray dashed lines are placed to better compare CCS profiles from the top profile to the bottom profile. All data was collected with the buffers in H_2O.

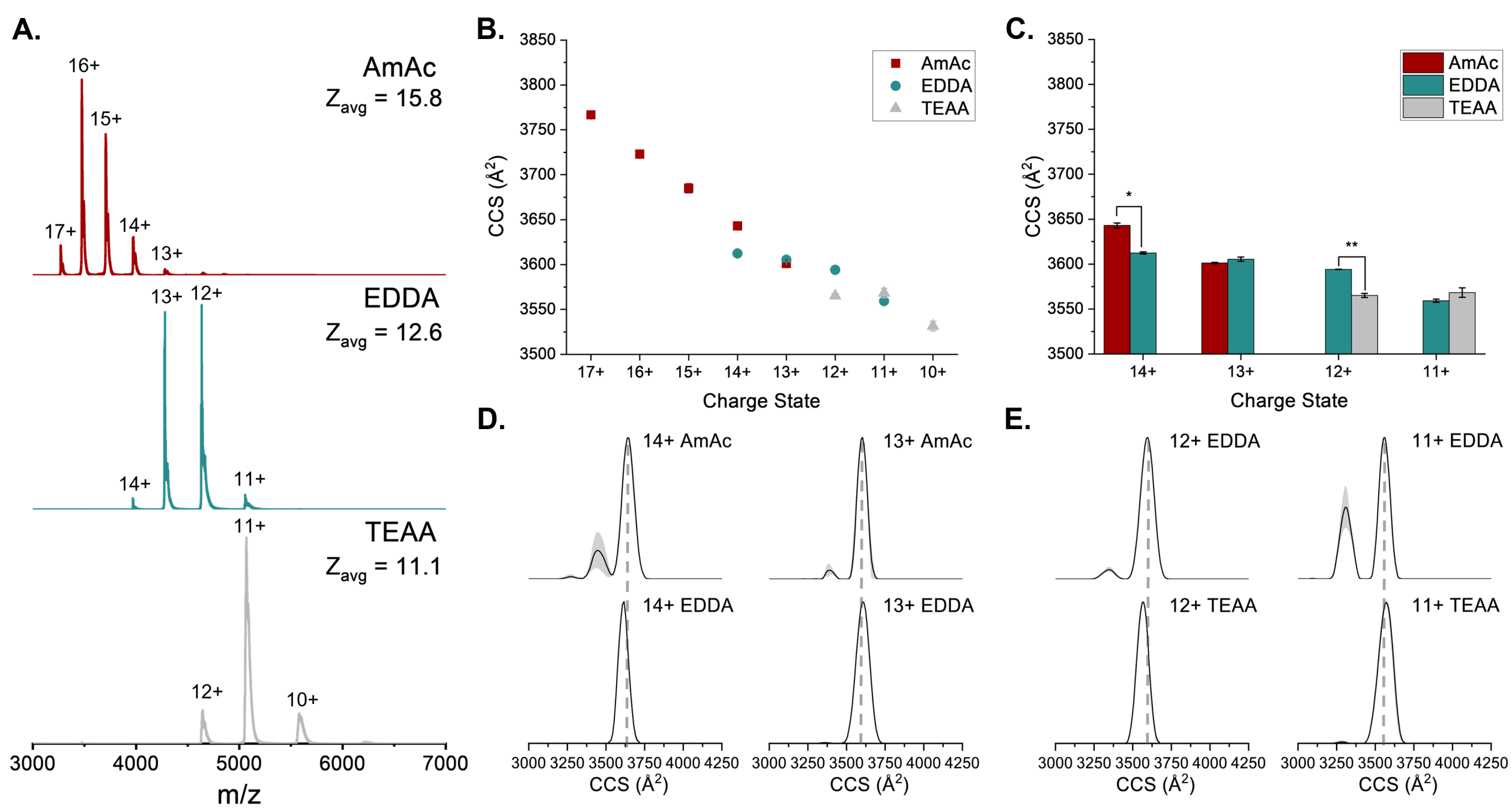

Figure S11: Native mass spectra and Z_{avg} of L55P in AmAc, EDDA, and TEAA **(A.)**. Scatter plot of the CCS of the most abundant conformer as a function of charge state for L55P in each buffer **(B.)**. Bar graph of the CCS for the most abundant conformer of L55P in overlapping charge states **(C.)**. CCS profiles of L55P for the 14+ and 13+ charge states of AmAc and EDDA **(D.)** along with the 12+ and 11+ charge states of L55P in EDDA and TEAA **(E.)**; shaded regions indicate standard deviation. Reported are the mean and standard deviation (n=3). Statistical significance as indicated: *p < 0.05, **p < 0.01 (paired two sample t-test, n = 3). Gray dashed lines are placed to better compare CCS profiles from the top profile to the bottom profile. All data was collected with the buffers in H_2O.

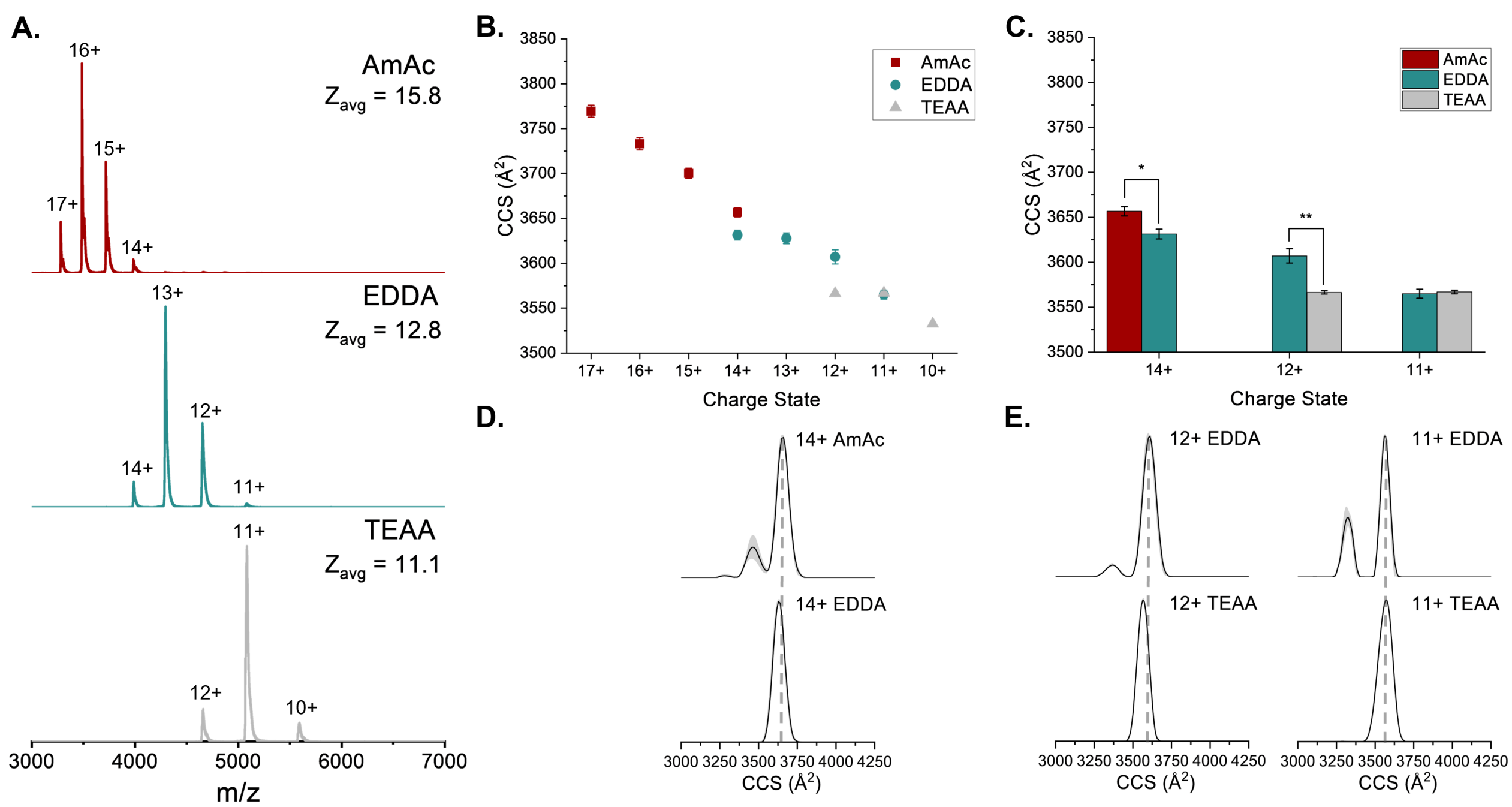

Figure S12: Native mass spectra and Z_{avg} of T119M in AmAc, EDDA, and TEAA **(A.)**. Scatter plot of the CCS of the most abundant conformer as a function of charge state for T119M in each buffer **(B.)**. Bar graph of the CCS for the most abundant conformer of T119M in overlapping charge states **(C.)**. CCS profiles of T119M for the 14+ charge state of AmAc and EDDA **(D.)** along with the 12+ and 11+ charge states of T119M in EDDA and TEAA **(E.)**; shaded regions indicate standard deviation. Reported are the mean and standard deviation (n=3). Statistical significance as indicated: *p < 0.05, **p < 0.01 (paired two sample t-test, n = 3). Gray dashed lines are placed to better compare CCS profiles from the top profile to the bottom profile. All data was collected with the buffers in H_2O.

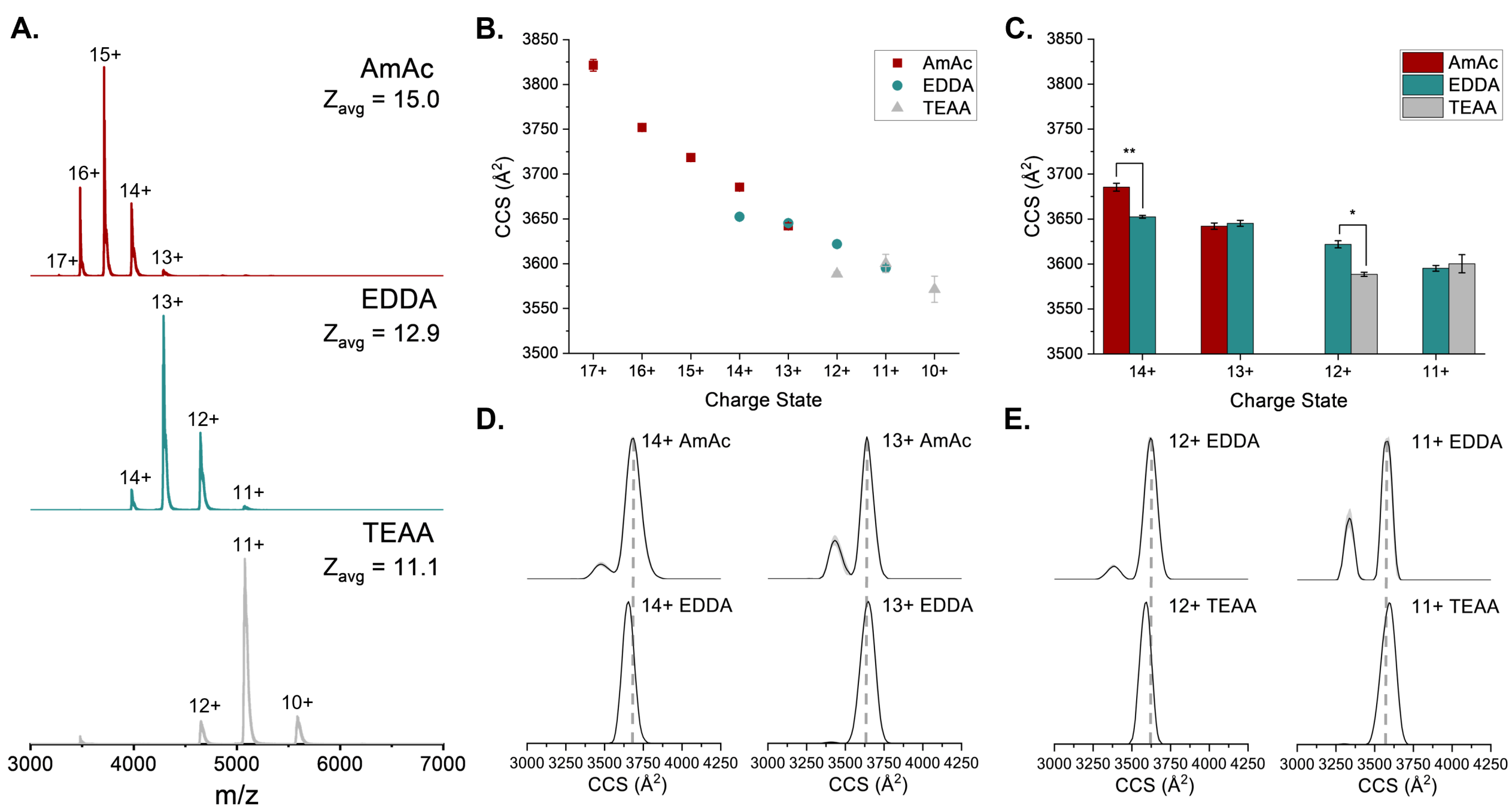

Figure S13: Native mass spectra and Z_{avg} of V122I in AmAc, EDDA, and TEAA **(A.)**. Scatter plot of the CCS of the most abundant conformer as a function of charge state for V122I in each buffer **(B.)**. Bar graph of the CCS for the most abundant conformer of V122I in overlapping charge states **(C.)**. CCS profiles of V122I for the 14+ and 13+ charge states of AmAc and EDDA **(D.)** along with the 12+ and 11+ charge states of L55P in EDDA and TEAA **(E.)**; shaded regions indicate standard deviation. Reported are the mean and standard deviation (n=3). Statistical significance as indicated: *p < 0.05, **p < 0.01 (paired two sample t-test, n = 3). Gray dashed lines are placed to better compare CCS profiles from the top profile to the bottom profile. All data was collected with the buffers in H_2O.

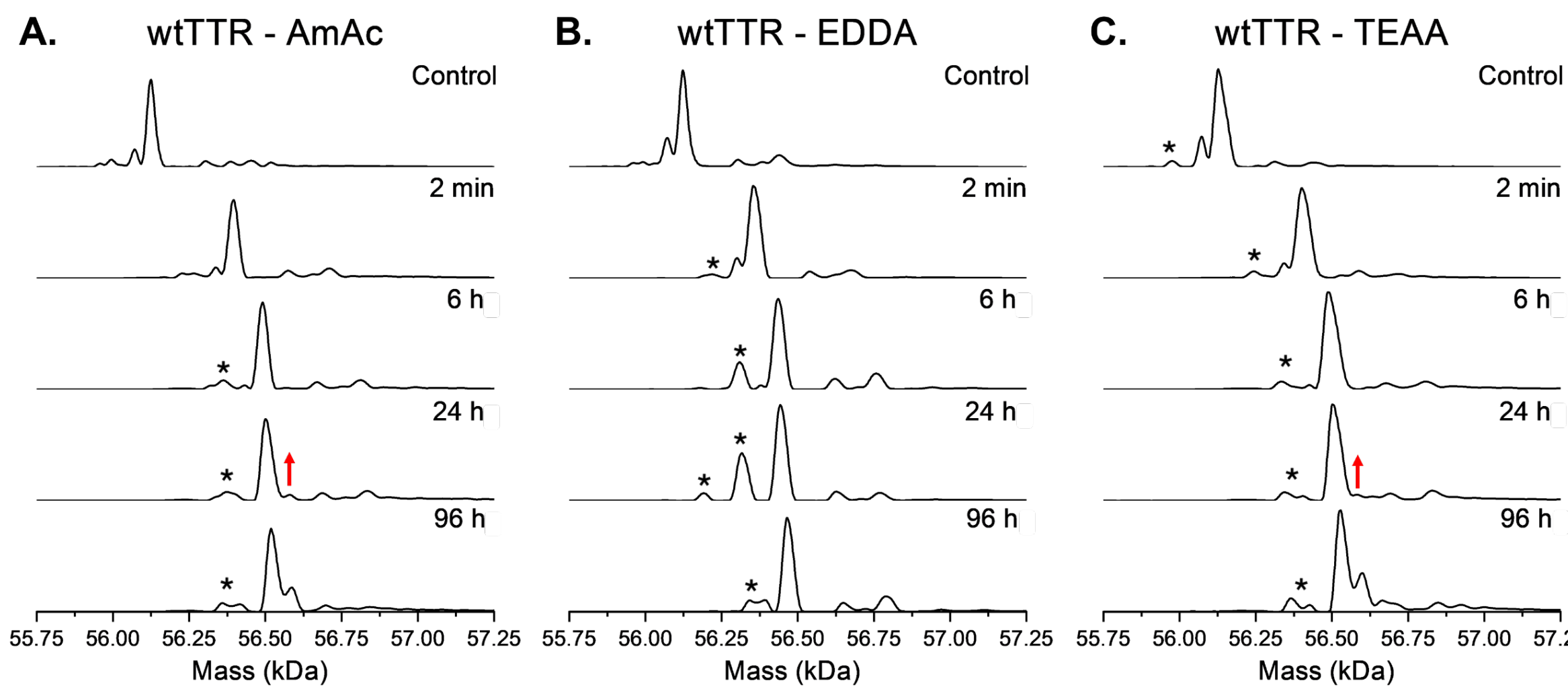

Figure S14: Deconvoluted mass showing mass shift over time after dilution of wtTTR with deuterium form of buffers AmAc **(A.)**, EDDA **(B.)**, and TEAA **(C.)**. * indicates different species of NEM bound to wtTTR rather than the 4 NEM bound peak used to determine %D exchanged (most abundant peak). Red arrows point to the increase of a second, higher mass peak indicative of tetramer disassembly and reassembly.

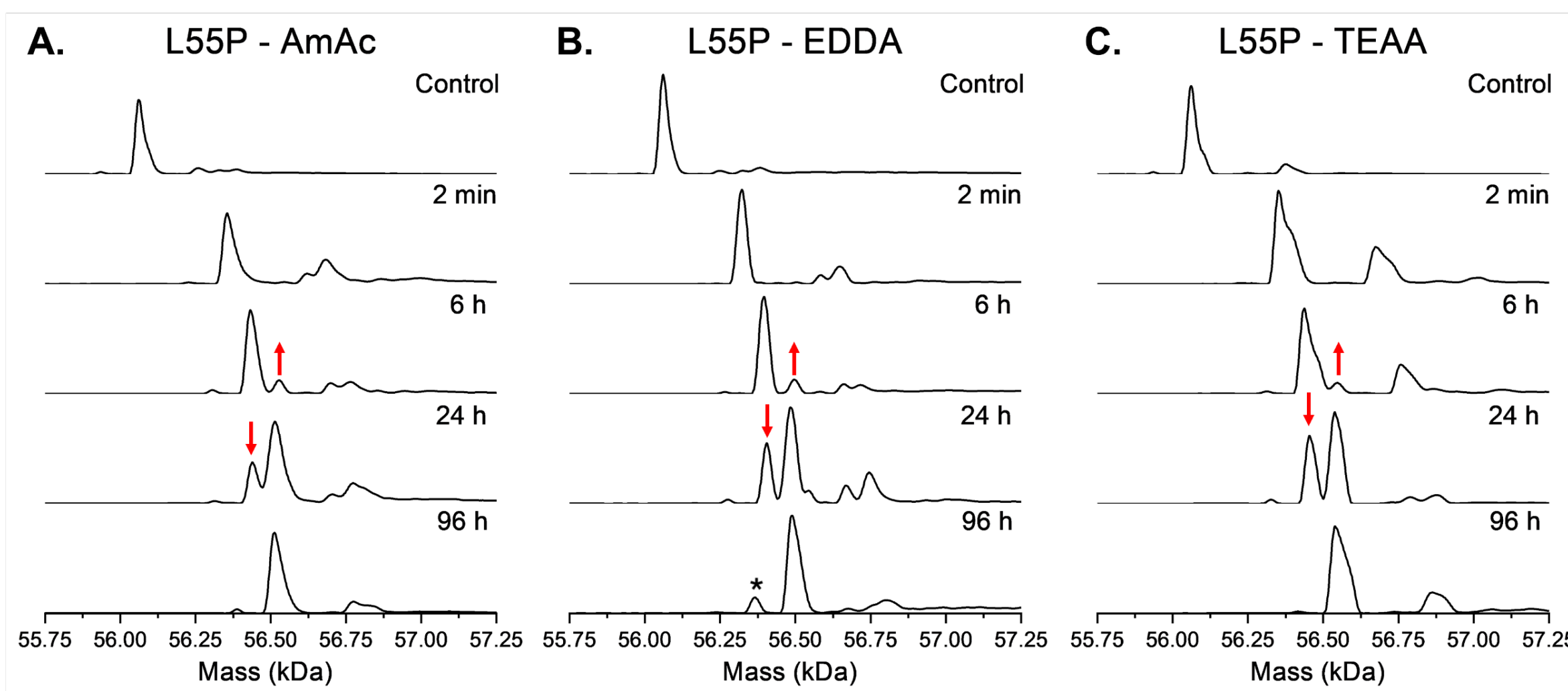

Figure S15: Deconvoluted mass showing mass shift over time after dilution of L55P with deuterium form of buffers AmAc **(A.)**, EDDA **(B.)**, and TEAA **(C.)**. * indicates different species of NEM bound to wtTTR rather than the 4 NEM bound peak used to determine %D exchanged (most abundant peak). Red arrows point to the increase of a second, higher mass peak and decrease of the original mass peak, indicative of tetramer disassembly and reassembly.

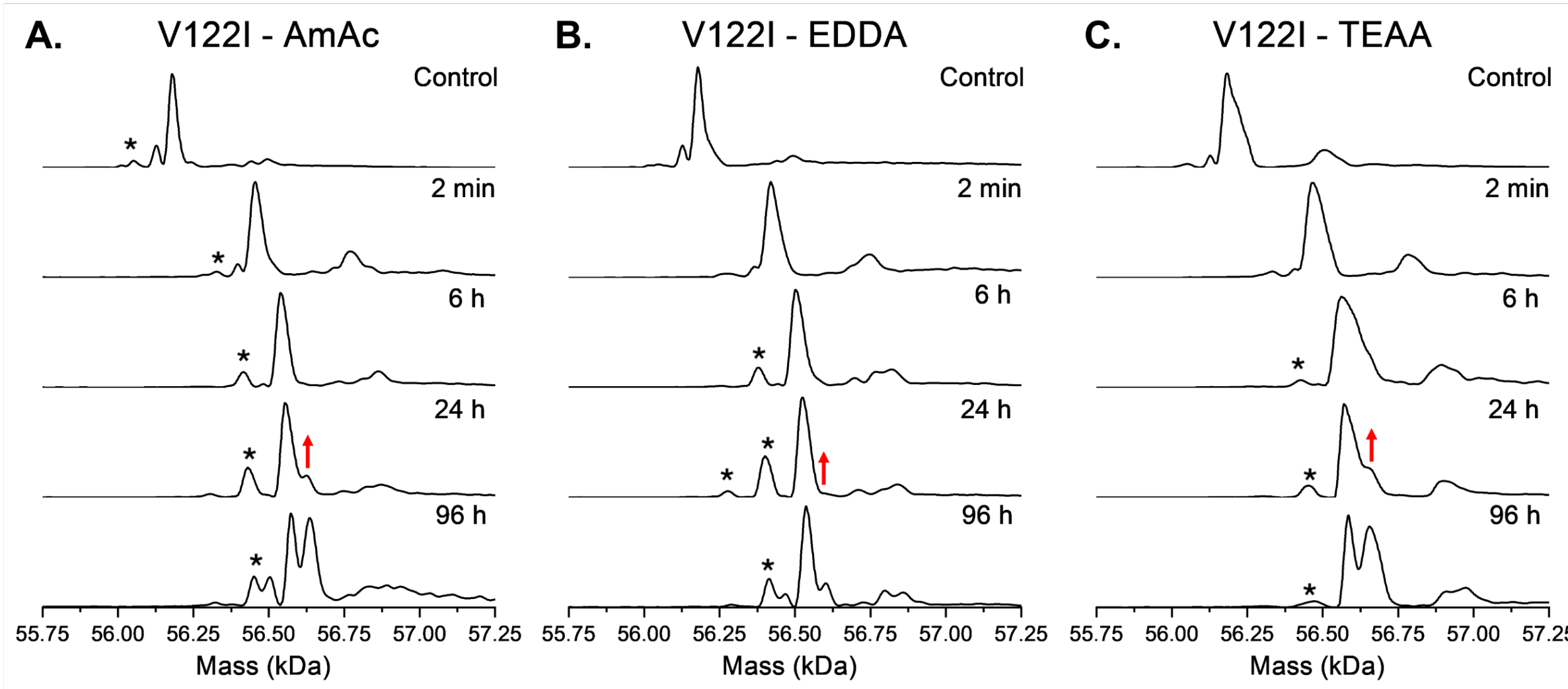

Figure S16: Deconvoluted mass showing mass shift over time after dilution of V122I with deuterium form of buffers AmAc **(A.)**, EDDA **(B.)**, and TEAA **(C.)**. * indicates different species of NEM bound to wtTTR rather than the 4 NEM bound peak used to determine %D exchanged (most abundant peak). Red arrows point to the increase of a second, higher mass peak indicative of tetramer disassembly and reassembly.

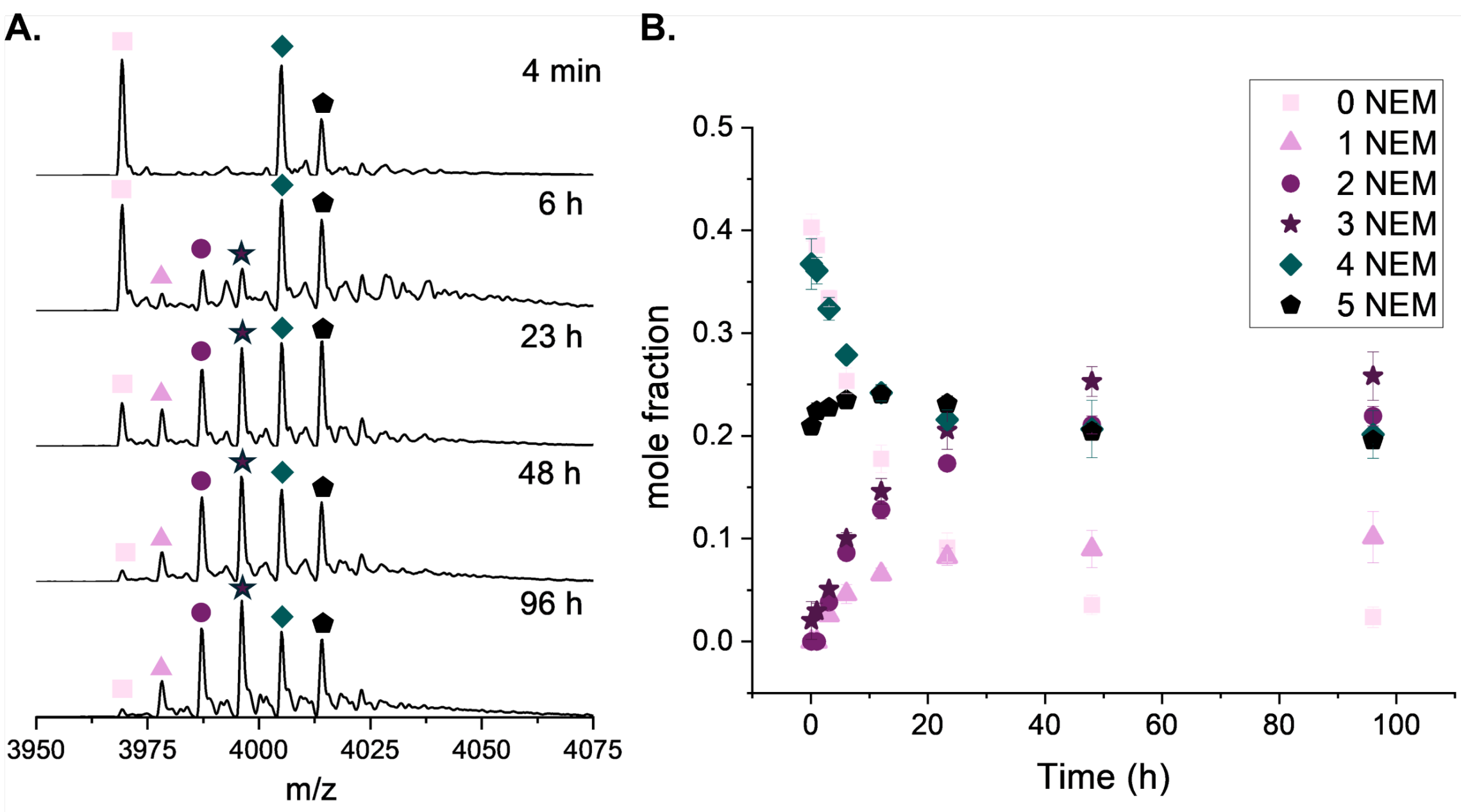

Figure S17: Close up of the 14+ charge state of L55P in 200 mM AmAc looking at different NEM-bound states on the protein complex. Spectra show how NEM distribution shifts over time, eventually reaching equilibrium **(A.)** Mole fraction plot of each NEM bound state of L55P. Data represents average and standard deviation (n = 3) **(B.)**. Since there are only 4 cysteines in total for the TTR tetramer, only 4NEM-bound species would be expected. However, due to a long incubation (~24 h) it appears a 5th NEM nonspecifically bound to TTR as well.

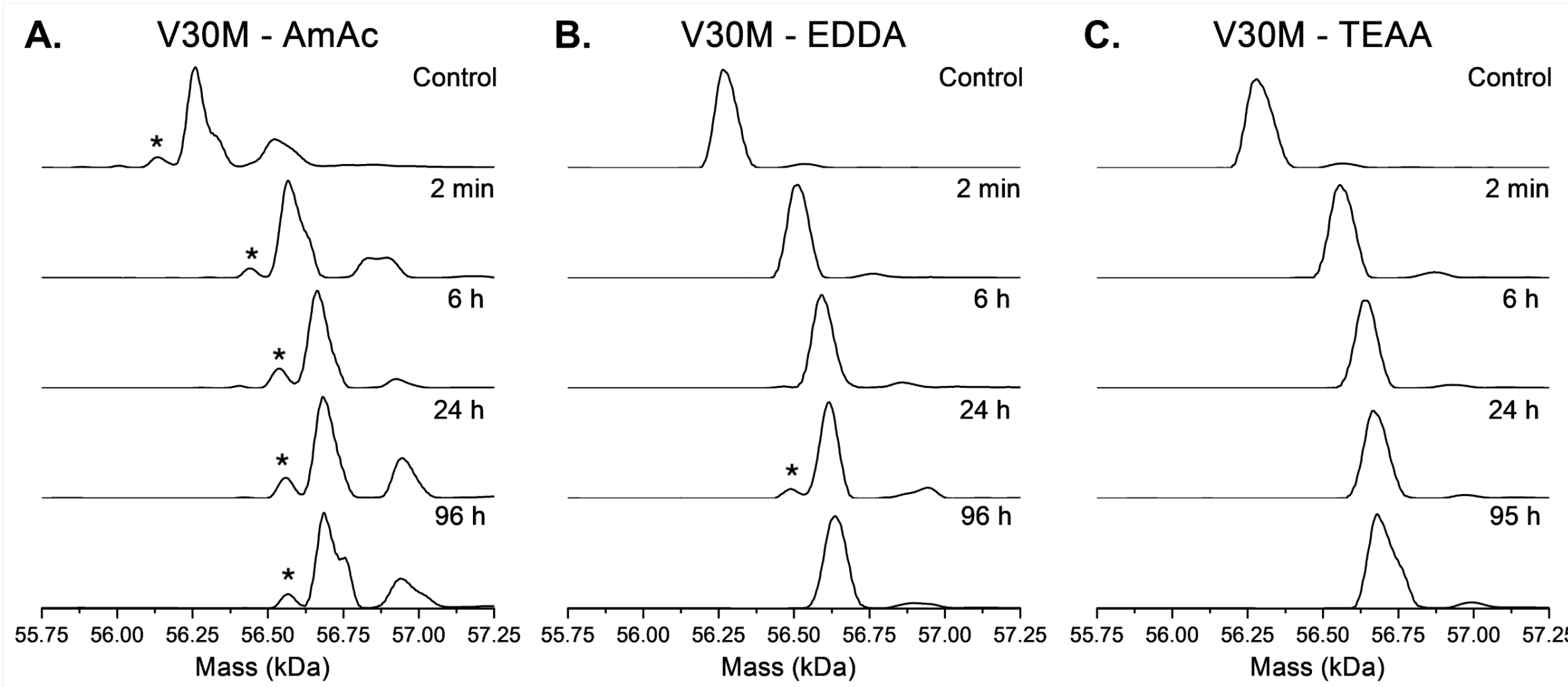

Figure S18: Deconvoluted mass showing mass shift over time after dilution of V30M with deuterium form of buffers AmAc **(A.)**, EDDA **(B.)**, and TEAA **(C.)**. * indicates different species of NEM bound to wtTTR rather than the 4 NEM bound peak used to determine %D exchanged (most abundant peak). After 96 h in AmAc and TEAA, there is a shoulder to the right of the mass peak. However, due to low resolution and the presence of adducts, it is not immediately obvious if this could be indicative of tetramer disassembly and reassembly.

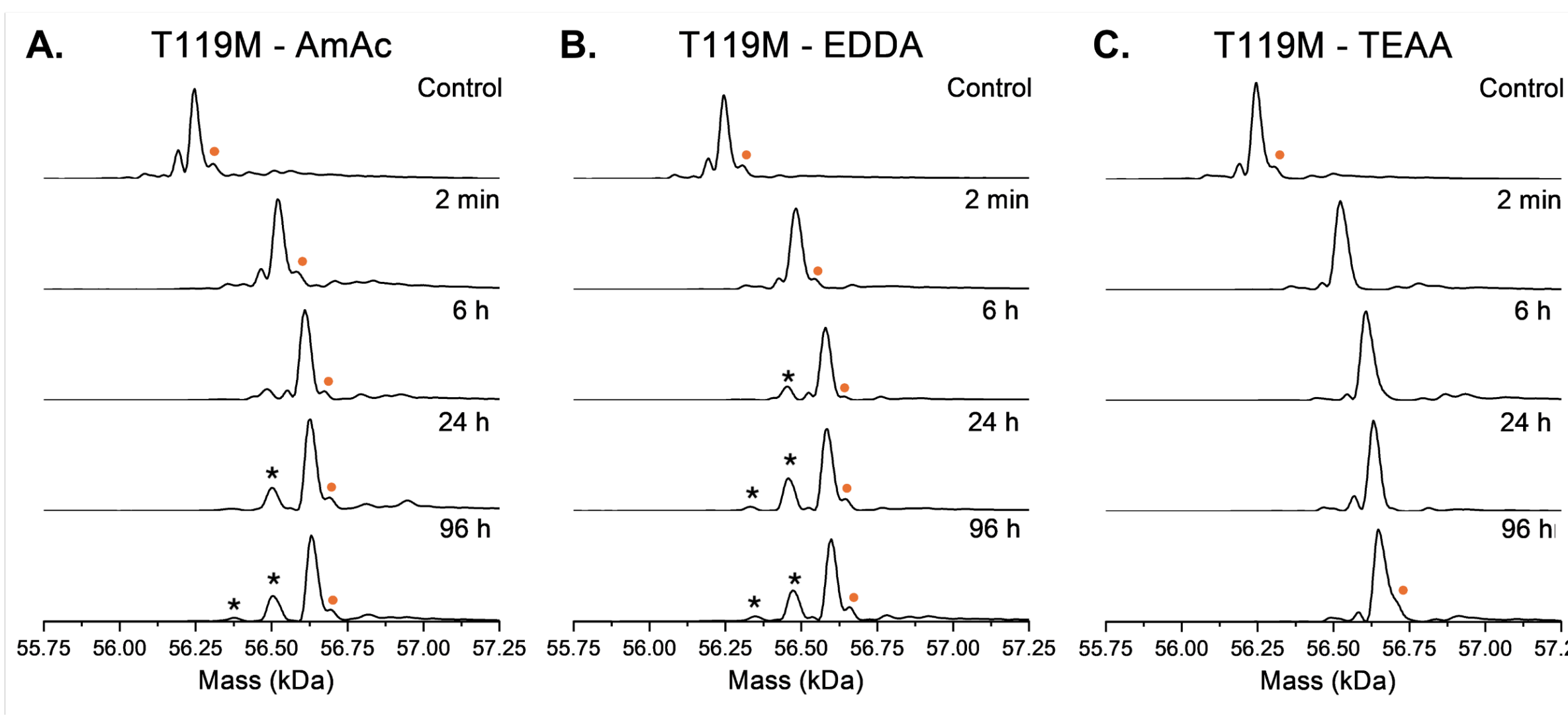

Figure S19: Deconvoluted mass showing mass shift over time after dilution of T119M with deuterium form of buffers AmAc **(A.)**, EDDA **(B.)**, and TEAA **(C.)**. * indicates different species of NEM bound to wtTTR rather than the 4 NEM bound peak used to determine %D exchanged (most abundant peak). Orange circle represents an adduct peak, most likely a Zn or Cu.

Table S1: Full width at half maximum (FWHM) of the CCS for the most abundant conformer of each proteoform in each buffer.

Proteoform	Buffer	Charge State	FWHM (Å^2)	Proteoform	Buffer	Charge State	FWHM (Å^2)
wtTTR	AmAc	17+	127 ± 6	L55P	EDDA	13+	98.5 ± 1.6
wtTTR	AmAc	16+	127 ± 9	L55P	EDDA	12+	99.6 ± 1.8
wtTTR	AmAc	15+	113 ± 8	L55P	EDDA	11+	80.5 ± 3.4
wtTTR	AmAc	14+	98.6 ± 5.1	L55P	TEAA	12+	87.6 ± 2.9
wtTTR	AmAc	13+	88.9 ± 5.2	L55P	TEAA	11+	106 ± 3
wtTTR	EDDA	14+	83.6 ± 5.4	L55P	TEAA	10+	80.2 ± 2.1
wtTTR	EDDA	13+	103 ± 7	T119M	AmAc	17+	134 ± 3
wtTTR	EDDA	12+	97.2 ± 3.1	T119M	AmAc	16+	126 ± 2
wtTTR	EDDA	11+	80 ± 3	T119M	AmAc	15+	105 ± 1
wtTTR	TEAA	12+	82.0 ± 5.2	T119M	AmAc	14+	91 ± 1
wtTTR	TEAA	11+	97.9 ± 5.9	T119M	EDDA	14+	87.0 ± 2.8
wtTTR	TEAA	10+	77 ± 4	T119M	EDDA	13+	106 ± 4
V30M	AmAc	17+	134 ± 4	T119M	EDDA	12+	96.7 ± 1.8
V30M	AmAc	16+	129 ± 4	T119M	EDDA	11+	68.8 ± 5.8
V30M	AmAc	15+	115 ± 6	T119M	TEAA	12+	86.0 ± 2.2
V30M	AmAc	14+	98.9 ± 4.4	T119M	TEAA	11+	105 ± 1
V30M	AmAc	13+	84.6 ± 3.1	T119M	TEAA	10+	83.6 ± 1.5
V30M	EDDA	14+	85.4 ± 3.7	V122I	AmAc	17+	78.3 ± 17.8
V30M	EDDA	13+	102 ± 8	V122I	AmAc	16+	138 ± 1
V30M	EDDA	12+	95.9 ± 4.6	V122I	AmAc	15+	134 ± 2
V30M	EDDA	11+	81.3 ± 1.9	V122I	AmAc	14+	112 ± 1
V30M	TEAA	12+	87.4 ± 0.9	V122I	AmAc	13+	91 ± 2
V30M	TEAA	11+	106 ± 1	V122I	EDDA	14+	89.5 ± 1.5
V30M	TEAA	10+	85.3 ± 1.9	V122I	EDDA	13+	107 ± 1
L55P	AmAc	17+	128 ± 2	V122I	EDDA	12+	94.1 ± 0.8
L55P	AmAc	16+	125 ± 3	V122I	EDDA	11+	55.6 ± 7.2
L55P	AmAc	15+	102 ± 2	V122I	TEAA	12+	85.4 ± 1.6
L55P	AmAc	14+	88.9 ± 0.5	V122I	TEAA	11+	91.9 ± 15.7
L55P	AmAc	13+	74.0 ± 8.9	V122I	TEAA	10+	67.7 ± 20.3
L55P	EDDA	14+	76.5 ± 2.1				

Table S2: *p*-values at 48 h for intact protein HDX experiments across all proteoforms and buffers. Yellow highlighted values indicate $p < 0.05$, orange highlighted values indicate $p < 0.01$ (paired two sample t-test, n = 3).

Proteoform 1	Proteoform 2	*p* value - AmAc	*p* value - EDDA	*p* value - TEAA
WT	V30M	0.0176	0.00427	0.115
WT	L55P	0.725	0.0516	0.721
WT	T119M	0.216	0.126	0.115
WT	V122I	0.438	0.0499	0.157
L55P	V30M	0.00538	0.0754	0.0647
L55P	T119M	0.304	0.0304	0.456
L55P	V122I	0.931	0.172	0.865
T119M	V30M	0.0102	0.00645	0.0103
T119M	V122I	0.0228	0.106	0.142
V30M	V122I	0.0194	0.0369	0.0301

Table S3: *p*-values of CCS_{avg} across all proteoforms and buffers. Yellow highlighted values indicate $p < 0.05$, orange highlighted values indicate $p < 0.01$ (paired two sample t-test, n = 3).

Proteoform 1	Proteoform 2	*p* value - AmAc	*p* value - EDDA	*p* value - TEAA
WT	V30M	0.133	0.543	0.322
WT	L55P	0.00109	0.0132	0.0265
WT	T119M	0.437	0.132	0.0296
WT	V122I	0.098	0.708	0.215
L55P	V30M	0.0285	0.0115	0.015
L55P	T119M	0.213	0.00965	0.698
L55P	V122I	0.0298	0.00331	0.00719
T119M	V30M	0.777	0.0551	0.0208
T119M	V122I	0.958	0.0683	0.00101
V30M	V122I	0.745	0.440	0.0962
